# CeiTEA: Adaptive Hierarchy of Single Cells with Topological Entropy

**DOI:** 10.1002/advs.202503539

**Published:** 2025-04-17

**Authors:** Bowen Tan, Shiying Li, Mengbo Wang, Shuai Cheng Li

**Affiliations:** ^1^ City University of Hong Kong Shenzhen Research Institute; ^2^ Department of Computer Science City University of Hong Kong Kowloon Hong Kong

**Keywords:** entropy, hierarchical clustering, single cell

## Abstract

Advances in single‐cell RNA sequencing (scRNA‐seq) enable detailed analysis of cellular heterogeneity, but existing clustering methods often fail to capture the complex hierarchical structures of cell types and subtypes. CeiTEA is introduced, a novel algorithm for adaptive hierarchical clustering based on topological entropy (TE), designed to address this challenge. CeiTEA constructs a multi‐nary partition tree that optimally represents relationships and diversity among cell types by minimizing TE. This method combines a bottom‐up strategy for hierarchy construction with a top‐down strategy for local diversification, facilitating the identification of smaller hierarchical structures within subtrees. CeiTEA is evaluated on both simulated and real‐world scRNA‐seq datasets, demonstrating superior clustering performance compared to state‐of‐the‐art tools like Louvain, Leiden, K‐means, and SEAT. In simulated multi‐layer datasets, CeiTEA demonstrated superior performance in retrieving hierarchies with a lower average clustering information distance of 0.15, compared to 0.39 from SEAT and 0.67 from traditional hierarchical clustering methods. On real datasets, the CeiTEA hierarchy reflects the developmental potency of various cell populations, validated by gene ontology enrichment, cell‐cell interaction, and pseudo‐time analysis. These findings highlight CeiTEA's potential as a powerful tool for understanding complex relationships in single‐cell data, with applications in tumor heterogeneity and tissue specification.

## Graphical abstract

CeiTEA, a novel hierarchical clustering algorithm based on topological entropy, effectively captures complex structures underlying data. By constructing an adaptive multi‐nary partition tree, CeiTEA reveals hierarchical structures and local diversifications, outperforming existing methods in clustering accuracy and consistency and providing the potential as a powerful tool for understanding complex relationships in single‐cell data.

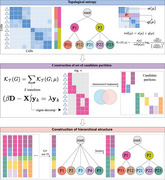

## 1 Introduction

Recent advances in single‐cell RNA sequencing (scRNA‐seq) and spatial transcriptomics (ST) have illuminated the intricate hierarchical organization of cell types and subtypes within biological systems.^[^ ^1^, ^2^ ^]^ These cell clusters exhibit varying depth and breadth, reflecting different levels of intra‐diversity influenced by developmental stages, homeostatic conditions, and environmental responses. Here, depth refers to the number of hierarchical layers in a tree, representing vertical differentiation among cell types and subtypes, with deeper hierarchies capturing more complex relationships. Breadth refers to the number of child nodes branching from a parent node, indicating horizontal diversity or heterogeneity, with broader hierarchies reflecting greater intra‐diversity at a given level. For instance, pluripotent stem cells exhibit a higher level of intra‐diversity and, potentially, a greater breadth in the hierarchy due to their extensive differentiation potential, allowing them to generate nearly any cell type in the body. In contrast, multipotent, oligopotent, and unipotent stem cells possess progressively narrower differentiation capabilities, which could result in their reduced breadth within the hierarchy.^[^ ^3^ ^]^ Furthermore, environmental factors, functional roles, and evolutionary diversification contribute to the heterogeneity within sub‐populations, influencing both their depth and breadth in the hierarchical structure. For example, T cell populations exhibit significant genetic diversity and adaptability, possibly with a broader breadth and depth compared to more functionally conserved B cell populations, which tend to have narrower differentiation capabilities and less intra‐diversity.^[^ ^4^ ^]^ In ST data, cell regions exhibit hierarchical structures characterized by broader spatial domains that may encompass varying numbers of subregions with different depths.^[^ ^5^ ^]^ Consequently, constructing a hierarchy that adapts to cellular diversity can enhance our understanding of the relationships among cell types and subtypes. Both the depth and breadth of a cell cluster should serve as indicators of its diversity within the global hierarchy.

However, existing methods provide insufficient support for analyzing these hierarchical structures, as they often impose rigid constraints, such as binary or balanced tree structures, which may not accurately capture the adaptive nature of biological hierarchies. Common clustering methods employed in scRNA‐seq, such as Louvain and Leiden, often yield flat, 2D representations that may overlook the complexities of cellular relationships.^[^ ^6^ ^]^ Current practices for studying multi‐level heterogeneity typically involve generating a fixed number of clusters and then adjusting the clustering resolutions in an ad hoc manner.^[^ ^7^ ^]^ For example, MRtree represents flat clustering results across different resolutions as a multi‐partite graph, constructing a binary tree that maintains the original hierarchical order established by the flat clustering algorithm.^[^ ^8^ ^]^ However, such ad hoc approaches risk losing critical hierarchical information and require multiple rounds of re‐clustering to determine an appropriate resolution. Similarly, ST data segmentation methods extract simplistic regions, ignoring important subregions that may have distinct biological significance.^[^ ^9^ ^]^

As an alternative, hierarchical clustering allows for direct multi‐resolution exploration of hierarchical cell heterogeneity. However, traditional hierarchical clustering algorithms are often limited to small datasets due to their high computational complexity. Approaches developed for large‐scale scRNA‐seq data, such as CellBIC and SEAT, rely on a binary tree model, which restricts their ability to capture meaningful depth and breadth.^[^ ^10^, ^11^ ^]^ These methods are inadequate for representing one‐to‐many and many‐to‐many relationships between cell types and subtypes.

In contrast, an adaptive hierarchy enables the construction of trees without such constraints, capturing meaningful variations in node depth and breadth that reflect the intra‐diversity and relationships among cell types. Here, we present CeiTEA, a method that constructs an adaptive hierarchy without constraints. We introduce a novel measure termed topological entropy (TE), which transcends traditional structural entropy by incorporating hierarchical relationships among individual nodes. This innovative approach facilitates the creation of a minimal TE hierarchy by iterating a single‐layer partition that minimizes TE through eigen‐decomposition and linear programming. Therefore, CeiTEA constructs an adaptive hierarchy that optimally represents cell‐type relationships and provides a rooted, unbalanced multi‐nary tree where the depth and breadth of internal nodes reflect their diversity.

## 2 Experimental Section

### 2.1 Overview of CeiTEA

CeiTEA introduces a novel entropy measure termed topological entropy (TE) considering the intricate hierarchical structure of cell types and subtypes. This measure differs from traditional structural entropy (SE) approaches: while structural entropy primarily considers the relationship between a node and its parent and external nodes, topological entropy emphasizes the local connectivity and provides a finer‐grained evaluation of the local topology of a graph. Utilizing this innovative entropy measure, CeiTEA aims to create a minimal TE hierarchy with an adaptive hierarchy construction process. This process begins with the generation of a TE‐minimizing single‐layer partition using Eigen decomposition and linear programming, followed by the adaptive layer construction through the iterative application of the single‐layer partitioning on supergraphs, where the partitions serve as leaves. CeiTEA optionally expands the hierarchy with a local diversification step, generating sub‐graphs from one of the partitions.

**Figure 1:**
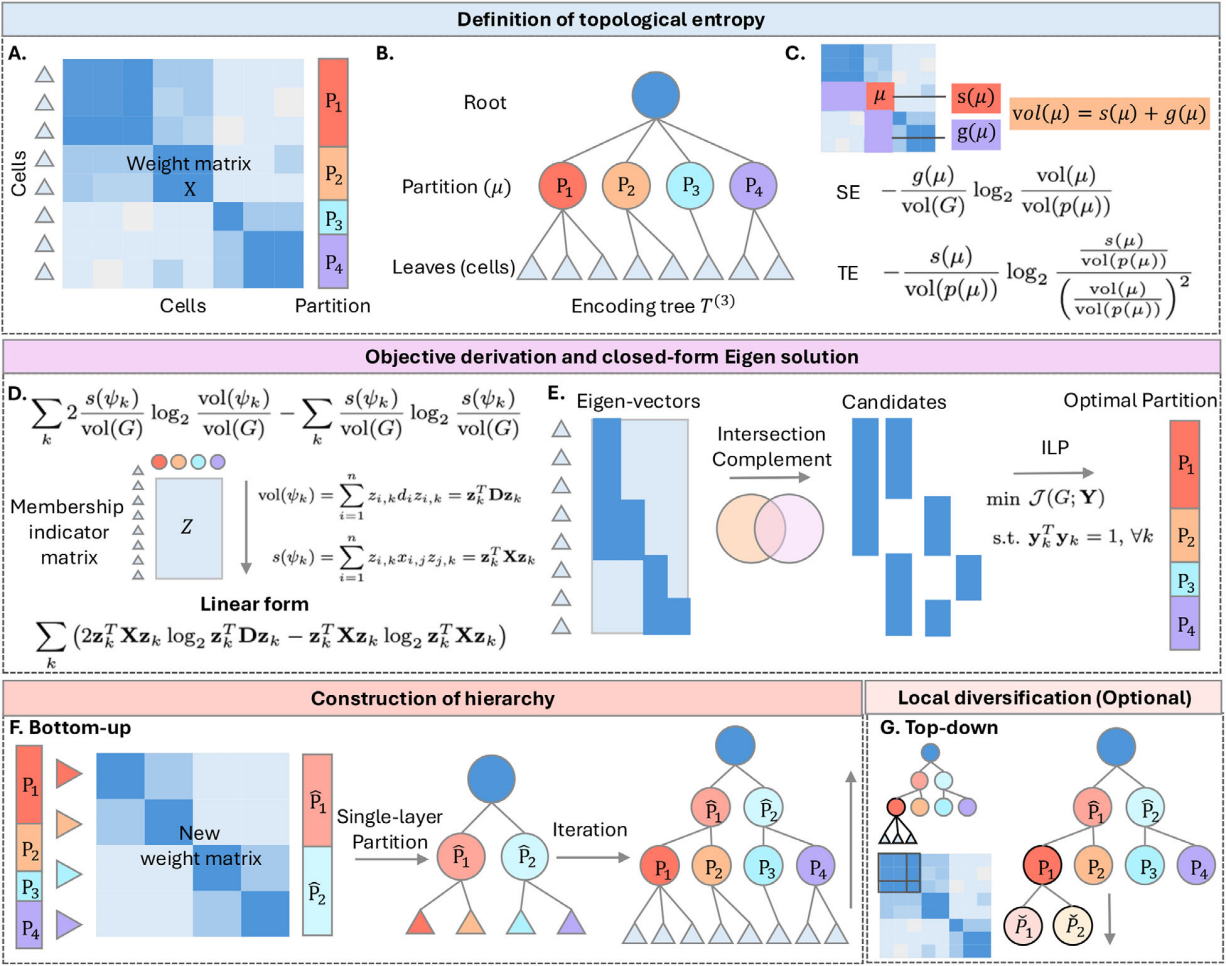
Workflow of CeiTEA. A–C) Definition of topological entropy. A) Input for structural/topological entropy: a cell‐cell weight matrix and a partition. B) Encoding tree of the given partition. C. Formulation of structural and topological entropy. D–E) Generation of a single‐layer partition with minimal topological entropy. D) Objective formulation to a linear form using a membership indicator matrix. E) Left: Candidate generation from the Eigen solution with intersections and complements; right: selection of the optimal partition with integer linear programming. F,G) Construction and optional reconstruction of hierarchy. F) Bottom‐up hierarchy construction by applying single‐layer partition on a super graph that treats partitions as new leaves. G) Optional top‐down hierarchy reconstruction for local diversification by applying single‐layer partition on a sub‐graph from one of the partitions.

Entropy is a widely recognized metric for evaluating the quality of a partition. In particular, considering a graph with an adjacency matrix as the similarity among cells, the structural entropy of a given partition reveals the degree of disorder or uncertainty by considering both the in‐degree and out‐degree of the partitions (**Figure** **1**A). A common definition of SE is through an encoding tree, also known as a partition tree.^[^ ^12^ ^]^ Specifically, a single‐layer partition can be conceptualized as a three‐layer hierarchical structure, where the leaves represent graph nodes, the internal nodes correspond to the actual partitions, and the root encapsulates the entire graph (Figure 1B). However, SE does not inherently account for the significance of different levels of a hierarchy structure. Therefore, we introduce topological entropy, which incorporates the hierarchical nature of the partition tree, reflecting how the partitions relate to their parent and child nodes (Figure 1C).

CeiTEA generates a single‐layer partition by minimizing TE with the following problem formulation (Figure 1D). Initially, we define a binary indicator matrix to represent the membership of vertices within various partitions. Each entry in this matrix indicates whether a vertex belongs to a specific partition. Concurrently, we define the objective function as TE across the partitions incorporating both the indicator matrix and the weight matrix. To optimize the objective function, we relax the binary constraints to a continuous space for a more flexible optimization landscape. Subsequently, we derive an equivalent linear form of the optimization problem, revealing that solutions to the optimization problem can be efficiently obtained via eigendecomposition. CeiTEA utilizes a user‐determined β range and a predefined number *n*_*e*_ of eigenvectors to generate partition candidates (Figure 1E). Each eigenvector can be considered as a binary partition of nodes, and the intersections and complements of these partitions generate a comprehensive pool of partition candidates. Subsequently, CeiTEA applies integer linear programming (ILP) to refine the selection of the optimal partition set with the objective of minimizing the total topological entropy. The optimal partition set obtained from ILP represents a single‐layer structure with minimized TE.

To construct a hierarchical structure, CeiTEA utilizes a bottom‐up approach based on the single‐layer partition method described above. As illustrated in Figure 1F, CeiTEA reinterprets internal nodes as new leaves and recalculates a new weight matrix, where each row and column corresponds to an internal node within the hierarchy. The single‐layer partitioning procedure is then applied to this new weight matrix, iterating until the partitioning stabilizes or results in two distinct partitions. At the conclusion of the iteration, CeiTEA generates a rooted, unbalanced multi‐nary tree, where the partition at each layer is designed to minimize topological entropy relative to their child nodes. Furthermore, the height of each internal node serves as an accurate indicator of the diversity within the overall tree, with nodes positioned lower in the hierarchy displaying greater diversity compared to those located closer to the root. CeiTEA optionally employs a top‐down strategy to reconstruct the hierarchy for local diversification, specifically to create additional subclusters, as illustrated in Figure 1G. Beginning at the root node, CeiTEA extracts the leaf nodes associated with each internal node, recalculates the weight matrix, and generates a single‐layer partition to form subclusters for that internal node. The resulting locally optimal multi‐nary tree replaces the original internal node, which may compromise the global hierarchy's optimality. This top‐down process proceeds along each branch until the TE of the partition no longer yields a negative value, thereby ensuring a valid hierarchical representation. Through this optional approach, CeiTEA achieves a hierarchy that is more attuned to local diversity, albeit at the cost of disrupting the globally TE‐optimal structure.

### 2.2 Entropy of an Encoding Tree

Given a graph (or network) *G* = (*V*, *E*; **X**) where $X\in R_{+}^{\left|V|\times |V\right|}$ is the symmetric non‐negative weight matrix of *G*, an *encoding tree* (or *partition tree*) T associated with *G* forms a hierarchical partitioning of the vertex set *V*. The root of the tree r(T) represents, or *encodes*, the whole vertex set *V*. For succinctness, we use *u* or *v* and μ or ν to represent a graph vertex and a tree node, respectively. Each tree node μ encodes a vertex subset *V*_μ_⊂*V*, and children of each tree node partition the vertices encoded by their parent node. Specially, each leaf node encodes a singleton vertex. Now we define the *volume* of a vertex set *V*_μ_ encoded by a tree node μ as $\mathrm{vol}\left(V_{\mu }\right)=\sum_{u\in V_{\mu },v\in V_{\mu }}x_{u,v}$ where $x_{u,v}$ is the edge weight between vertices *u* and *v*, and the *egress* of the same vertex set as $g\left(V_{\mu }\right)=\sum_{u\in V_{\mu },v\notin V_{\mu }}x_{u,v}$. In simplicity, we further denote vol(μ) = vol(*V*_μ_) and *g*(*V*_μ_) = *g*(μ). Let *p*(μ) denote the parent node of μ and $H_{T}\left(G;\mu \right)$ denote the *structural entropy* (SE) of node μ∈T. Then, the SE of the encoding tree T, $H_{T}\left(G\right)$, is computed as the sum of SE of all nodes in T except for the root,^[^ ^12^ ^]^ i.e.,

(1) $$\begin{array}{c} H_{T}\left(G\right)=\sum_{\mu \in T,\mu \neq r(T)}H_{T}\left(G;\mu \right)=\sum_{\mu \in T,\mu \neq r(T)}-\frac{g(\mu )}{\mathrm{vol}(G)}\log_{2}\frac{\mathrm{vol}(\mu )}{\mathrm{vol}(p(\mu ))}. \end{array}$$

While structural entropy defined above focuses on hierarchical parent‐to‐child transitions, we introduced and applied a revised version of structural entropy, termed *topological entropy* (TE), that incorporates the internal connectivity of subgraphs and local structural details.

Formally, for the given partition tree T, we define *s*(μ) = vol(μ) − *g*(μ) as the *cohesion* of the vertex set *V*_μ_, i.e., the $s\left(\mu \right)=s\left(V_{\mu }\right)=\sum_{u\in V_{\mu },v\in V_{\mu }}x_{u,v}$. Then, the total TE of all tree nodes except for the root is the TE of the whole encoding tree, $K_{T}\left(G\right)$, i.e.,

(2) $$\begin{array}{c} K_{T}\left(G\right)=\sum_{\mu \in T,\mu \neq r(T)}K_{T}\left(G;\mu \right)=\sum_{\mu \in T,\mu \neq r(T)}-\frac{s(\mu )}{\mathrm{vol}(p(\mu ))}\log_{2}\frac{\frac{s(\mu )}{\mathrm{vol}(p(\mu ))}}{{\left(\frac{\mathrm{vol}(\mu )}{\mathrm{vol}(p(\mu ))}\right)}^{2}} \end{array}$$

where $K_{T}\left(G;\mu \right)$ denotes the TE of a tree node μ.

In Equation (2), the cohesion *s*(μ) quantifies the degree of connectivity among the vertices within the subset encoded by μ. A higher cohesion indicates that the vertices are more interconnected, reflecting a stronger internal structure. In particular, topological entropy emphasizes the cohesion and normalizes the cohesion by the volume of the parent node *p*(μ), reflecting the local connectivity of nodes in the context of their parent node and managing to achieve a structure that better evaluates hierarchical quality in terms of connectivity. Furthermore, topological entropy is suited for multi‐nary hierarchies as it puts emphasis on the distribution of connections within parent nodes.

### 2.3 Algorithm of CeiTEA

The CeiTEA algorithm is designed to minimize the topological entropy (TE) of a graph *G* = (*V*, *E*; **X**), with the focus on a plain partitioning $P=(P_{1},\cdots ,P_{K})$ or a three‐layer hierarchical structures $T^{\left(3\right)}$ where the internal nodes encode the actual partitions of leaf nodes. By rescaling the weight matrix **X** such that vol(*G*) = ∑_*u*, *v*_ *x*_*u*, *v*_ = 1 and assuming a binary indicator matrix **Z** = (*z*_*i*, *k*_) ∈ {0, 1}^*n* × *K*^ where *z*_*i*, *k*_ = 1 if the node *V*_*i*_ ∈ ψ_*k*_ and ψ_*k*_ encodes *P*_*k*_, we can transform Equation (2) for $T^{\left(3\right)}$ to a matrix form, that is

(3) $$\begin{array}{c} K_{T^{\left(3\right)}}\left(G,P\right)=\sum_{k}\left(2z_{k}^{T}Xz_{k}\log_{2}z_{k}^{T}Dz_{k}-z_{k}^{T}Xz_{k}\log_{2}z_{k}^{T}Xz_{k}\right) \end{array}$$

where **D** = diag(*d*_1_, …, *d*_*n*_) is the diagonal matrix with *d*_*i*_ as degrees or total weights associated with *V*_*i*_. Our goal is to minimize Equation (3). By relaxing **Z** to a continuous space $Y\in R^{n\times K}$, we can rewrite Equation (3) as J(G;Y), and applying Lagrangian multipliers α′*s* with constraints $y_{k}^{T}y_{k}=1$, we can derive the Lagrangian function L(G;Y) as

(4) $$\begin{array}{cc} J(G;Y) & =\sum_{k}\left(2y_{k}^{T}Xy_{k}\log_{2}y_{k}^{T}Dy_{k}-y_{k}^{T}Xy_{k}\log_{2}y_{k}^{T}Xy_{k}\right) \end{array}$$

(5) $$\begin{array}{cc} L(G;Y) & =J\left(G;Y\right)+\sum_{k}\alpha_{k}f_{k}\left(Y\right) \end{array}$$

where *f*_*k*_(**Y**) = **y** ^*T*^ **y** − 1 and hence to solve it we need to solve for each **y**_*k*_ such that

(6) $$\begin{array}{c} \nabla_{y_{k}}J=\alpha_{k}\nabla_{y_{k}}f_{k}\left(Y\right). \end{array}$$

By arranging terms, we can write Equation (6) for each **y**_*k*_ as

(7) $$\begin{array}{c} \left[\frac{2y_{k}^{T}Xy_{k}}{y_{k}^{T}Dy_{k}\ln2}D+\left(2\log_{2}y_{k}^{T}Dy_{k}-\log_{2}y_{k}^{T}Xy_{k}-\frac{y_{k}^{T}Xy_{k}}{y_{k}^{T}Xy_{k}\ln2}\right)X\right]y_{k}=\alpha_{k}y_{k}. \end{array}$$

Due to the complexity of the equation, it is challenging to obtain exact solutions for **y**_*k*_ and α_*k*_. Therefore, we adopted a heuristic approach to approximate the solutions. By introducing substitutions with β and λ (as given in Equation 11 of Supporting Information), Equation (7) can be transformed into

(8) $$\begin{array}{c} \left(\beta D-X\right)y_{k}=\lambda y_{k}. \end{array}$$

Instead of analytically solving for **y**_*k*_ that β depends on, we enumerate β from a predetermined discrete range based on the estimation of β values (see Supplementary Methods). It can be observed that **y**_*k*_ corresponds to an eigenvector of the eigendecomposition of β**D** − **X**. Hence, we can perform the eigendecomposition on the matrix **M**_β_ = β**D** − **X** for a given β and solve for the eigenvectors as the approximated solutions for **Y**. When β = 1, in addition, **y**_*k*_ is one of the eigenvectors of the Laplacian matrix of the graph *G* and the spectral clustering can be applied to perform the partitions. Here, we extend the concept that the Fiedler vector (corresponding to the first non‐zero eigenvalue) partitions the graph into two subgraphs to eigenvectors corresponding to the first *n*_*e*_ eigenvalues, where *n*_*e*_ is a predefined number. By eigendecomposition on each **M**_β_, we maintain a collection of partitions from eigenvectors. As true partitions may be obtained by intersecting and complementing among partitions in the collection (Figure S1A, Supporting Information), we can prepare a candidate indicator matrix $\hat{Z}=\left({\hat{z}}_{1},\cdots ,{\hat{z}}_{N}\right)\in {\left\{0,1\right\}}^{n\times N}$ from intersections and complements between pairs of partitions, which allows us to identify an optimal set of partitions with the minimized topological entropy.

Notably, each partition contributes independently to the topological entropy of $T^{\left(3\right)}$, suggesting an additive property and allowing the application of integer linear programming (ILP) techniques. For each ${\hat{z}}_{j}$, we compute the corresponding topological entropy as $e_{j}=2{\hat{z}}_{j}^{T}X{\hat{z}}_{j}\log_{2}{\hat{z}}_{j}^{T}D{\hat{z}}_{j}-{\hat{z}}_{j}^{T}X{\hat{z}}_{j}\log_{2}{\hat{z}}_{j}^{T}X{\hat{z}}_{j}$ and then denote **e** = (*e*_1_, …, *e*_*N*_)^*T*^. Let *b*_*j*_ ∈ {0, 1} be the indicator determining whether ${\hat{z}}_{j}$ is included in the optimal set. Hence, we can represent the total entropy of selected candidates as **b** ^*T*^ **e** where **b** = (*b*_1_, …, *b*_*N*_)^*T*^. As a result, the objective of CeiTEA in the ILP form is

(9) $$\begin{array}{cc} \min & \;b^{T}e\\ \text{s.t.} & \;\hat{Z}b=1 \end{array}$$

where **1** is a column vector with all entries as one. Eventually, columns of $\hat{Z}$ with *b* = 1 compose of the optimal set $P_{\text{opt}}$ of partitions that minimize the topological entropy given a range of β.

The number and quality of partitions in the optimal set is significantly influenced by the number of candidates involved in the ILP, which results from the range of β and the number *n*_*e*_ of eigenvectors used to partition the graph. The quality of partitions is highly related to the range of β. A suitable β range can generate partition candidates of high accuracy and consistency, and hence reliable optimal partition set. On the other hand, *n*_*e*_ controls the size of candidates and potentially determines whether the correct candidate could be included for the ILP. A moderate number for *n*_*e*_ should be chosen, as a small number (e.g., ⩽3) may be insufficient to construct a reliable candidate set, while a large number (e.g., ⩾20) may include eigenvectors that generate partitions with little significance or that are uninterpretable. As the graph size expands, however, the candidate set grows rapidly, leading to substantial increases in the time complexity of candidate intersections and complements as well as the ILP. This necessitated the adoption of a greedy algorithm in the implementation (Supplementary Methods) to maintain an acceptable level of complexity (as demonstrated by running time and memory usages in Figure S2, Supporting Information).

### 2.4 Construction of a Stratified Hierarchy

The optimal partition set $P_{\text{opt}}$ obtained in the previous section is a plain partitioning, or a three‐layer hierarchy. To build a hierarchical structure for the graph *G*, we can perform a bottom‐up strategy on the obtained $P_{\text{opt}}$. At first, $P_{\text{opt}}$ is equivalent to $T_{\text{opt}}^{\left(3\right)}$ where the leaves encode graph vertices, internal nodes encode the actual partitions with *b* = 1, and the root encodes the whole graph *G*. Now, we treat the internal nodes as new leaves and recompute a new weight matrix **X**′ where each row and column correspond to one internal node of $T_{\text{opt}}^{\left(3\right)}$ (or one partition in $P_{\text{opt}}$). Thus, we apply the same procedure yielding $P_{\text{opt}}$ on **X**′ to partition **X**′. This process repeats until the partitioning either stabilizes, remaining unchanged from the previous iteration, or results in two partitions. Ultimately, this yields a multi‐nary tree, denoted as $T_{\text{opt-bt}}$.

Following the construction of the optimal $T_{\text{opt-bt}}$, a top‐down approach can be additionally employed to iteratively expand the tree, thereby identifying smaller possible hierarchical structures. This procedure, which we refer to as *local diversification*, involves a systematic exploration of substructures within subtrees, facilitating a more nuanced understanding of underlying hierarchical relationships. For every child node μ, starting from the root node, we extract all leaf nodes encoded by μ, recompute the weight matrix, find the corresponding candidate set, solve the associated ILP for a locally optimal multi‐nary tree and replace μ with the new tree. The local diversification along each branch terminates when the associated entropy of the partition from ILP is no smaller than a predetermined value (e.g., zero) to guarantee adequate entropy and hierarchy. In this way, a more stratified hierarchy is produced.

### 2.5 Experimental Setup

#### 2.5.1 Data Simulations

We utilized stochastic block models (SBM)^[^ ^13^ ^]^ to generate multiple graphs with varying parameter configurations. As a summary, **Table** **1** shows the configurations of SBM simulations for single‐layer graphs. For hierarchical simulations, we applied SBM iteratively to generate nested graphs with a variable number of layers, ranging from two to six, while maintaining consistent configurations for the cluster definitions and edge noises. The only variable for each simulated case parameter was the number of first‐layer partitions, which was randomly chosen from two to one‐tenth of the number of vertices.

**Table 1:** Simulation configurations.

#Vertices	#Partitions	Strongly defined		Weakly defined		Edge noise
		Pr(edge w/i partition)	Pr(edge b/t partitions)	Pr(edge w/i partition)	Pr(edge b/t partitions)	
100	2/5/10	0.80∼1.00	0.01 ∼0.05	0.60 ∼0.80	0.05 ∼0.10	0/0.1
200	2/5/10/20	0.80∼1.00	0.01 ∼0.05	0.60 ∼0.80	0.05 ∼0.10	0/0.1
500	2/5/10/20/50	0.80∼1.00	0.01 ∼0.05	0.60 ∼0.80	0.05 ∼0.10	0/0.1
1000	2/5/10/20/50/100	0.80∼1.00	0.01 ∼0.05	0.60 ∼0.80	0.05 ∼0.10	0/0.1

#### 2.5.2 Method Configurations

CeiTEA replies on two hyper‐parameters: β and the number *n*_*e*_ of eigenvectors. In this work, we estimated the possible ranges of β for nine real‐world single‐cell datasets with golden annotations and enumerated β to inspect the partitions generated by eigenvectors from different β values (Supplementary Methods and Figure S3, Supporting Information). As a result, we determined the value range of β as discrete values from 0.01 to 2 with a step of 0.01, which were observed to be effective and efficient. The number *n*_*e*_ of eigenvectors with the smallest topological entropy values for each β was determined by the graph size: if *n* > 100, CeiTEA used eigenvectors providing partitions with 10 smallest topological entropy, otherwise, all eigenvectors were involved.

In our single‐layer simulations, we benchmarked CeiTEA against several advanced clustering methods, including Louvain, Leiden, K‐means, spectral clustering, and agglomerative clustering (Agglo) with four distinct linkages (single, complete, average, and Ward), as well as SEAT.^[^ ^14^ ^]^ For the multi‐layer simulations, we compared CeiTEA with hierarchical clustering (HC) methods utilizing the same four linkages, in addition to SEAT. Furthermore, our evaluation extended to both structural and topological entropy objectives for CeiTEA and SEAT. For SEAT, which incorporates an entropy‐minimizing objective and allows for a strategy parameter (either bottom‐up or top‐down), we selected the partitions from the configuration that produced the lowest entropy as the results. The remaining parameters of SEAT are consistent for both single‐layer and multi‐layer simulated datasets: we set the “precomputed” for “affinity,” “affinity” for “sparsification” and “100” for “max_k”. To ensure a fair comparison, we set the number of clusters for methods without automatic cluster detection to align with the cluster counts generated by both SEAT and CeiTEA. For the Leiden and Louvain methods, we set the number of neighbors as 15 and adjusted the resolution parameter to match the desired output number of clusters.

Since Louvain, Leiden, K‐means, spectral clustering, and Agglo with the ward linkage required a sample‐feature matrix as the input, in addition, we applied the non‐negative matrix factorization (NMF) on the similarity matrix by SBM to estimate a feature matrix. For each simulated case, we set the number of components in NMF as the number of partitions of the ground truth. For Agglo with single, complete, and average linkages, the “precomputed” metric was used.

#### 2.5.3 Evaluation Metrics

The primary metrics used to evaluate the clustering quality of all methods were the adjusted Rand Index (ARI)^[^ ^15^ ^]^ and adjusted mutual information (AMI).^[^ ^16^ ^]^ Additionally, we employed the clustering information distance (CID) metric^[^ ^17^ ^]^ to assess the similarity between the true and estimated hierarchies produced by different methods. Moreover, metrics were aggregated for methods without automatic cluster detection; that is, for each of these methods, we averaged ARI and AMI scores across clustering outcomes under both SEAT and CeiTEA cluster numbers.

#### 2.5.4 Preprocessing on Spatial Transcriptome Datasets

**Figure 2:**
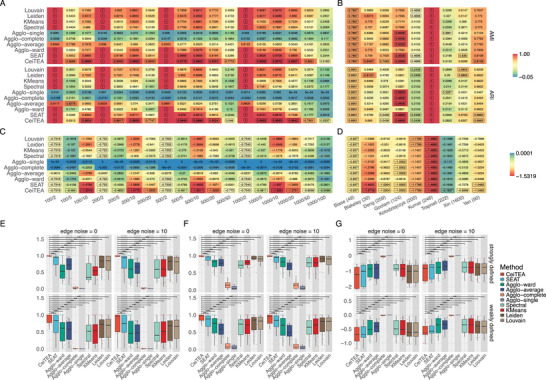
Evaluations of clustering performance with adjusted Rand Index (ARI) scores, adjusted mutual information (AMI) scores and topological entropy values of CeiTEA and other tools on single‐layer simulated datasets as well as single‐cell datasets. A,B) Heaptmaps of average ARI and AMI scores over different levels of edge noise and wellness of cluster definitions of all methods on simulated and single‐cell datasets. Values surrounded by boxes indicate maximum values among all methods. C,D) Heatmaps of average topological entropy values over different levels of edge noise and wellness of cluster definitions of all methods on simulated and single‐cell datasets. Values surrounded by boxes indicate minimum values among all methods. E) Comparisons of ARI scores across different levels of edge noise and wellness of cluster definitions. F) Comparisons of AMI scores across different levels of edge noise and wellness of cluster definitions. G) Comparisons of topological entropy values across different levels of edge noise and wellness of cluster definitions. The significance is calculated using a one‐sided paired Wilcoxon test, with the following annotations: **** for *p*‐value ⩽ 0.0001, *** for *p*‐value ⩽ 0.001, ** for *p*‐value ⩽ 0.01, * for *p*‐value ⩽ 0.05, and NS for *p*‐value > 0.05.

Spot annotations for the BC dataset are collected with the count matrices. For the PDAC dataset, the spot annotations are from **Figure** **2** in the original publication,^[^ ^18^ ^]^ and cell type deconvolution with paired scRNA‐seq data is performed by SPOTlight following the github tutorial. We used SpatialDE to identify spatially variable genes (SVGs) and SVG expression patterns in the spatial transcriptome data, using default parameters.^[^ ^19^ ^]^ SpatialDE models gene expression variability across spatial locations to distinguish truly spatially variable genes from those that vary only due to technical noise. We then clustered the SVG expression patterns using both Scanpy's Leiden and Louvain algorithms^[^ ^20^ ^]^ and CeiTEA. Leiden and Louvain were performed with the resolution parameter set to generate a predefined number of clusters, such as the number of predefined cell type annotations or tumor and non‐tumor annotations. The clustering results from Leiden, Louvain, and CeiTEA on the SVG patterns were compared to evaluate their performance in identifying spatial regions.

### 2.6 Downstream Analyses

#### 2.6.1 Gene Ontology Enrichment Analysis

We obtain the marker gene using Scanpy's “rank_genes_groups” function with respect to the CeiTEA clusters. We keep the top 50 differentially expressed genes for each cluster. Subsequently, we apply the “enrichR” function implemented in the GSEApy package to test for enrichment in the GO_Biological_Process_2023 database.^[^ ^21^ ^]^ We institute an adjusted *p*‐value cutoff at 0.05, a minimum number of involved genes at 2, and a minimum hit_ratio at 0.05, for calling significant enrichment.

#### 2.6.2 Cell‐to‐Cell Communications Analysis

The CellChat software was used to infer cell‐to‐cell interactions based on ligand‐receptor crosstalk.^[^ ^22^ ^]^ We filtered out cell‐cell communications that were expressed in fewer than three cells in any cell group. The “netVisual” function was used to visualize interaction hierarchy in selected receiver clusters. We focus on intra‐signaling, that is, signaling among cells within the same cluster. Furthermore, we removed the Laminim and Collagen pathways as they are present within most clusters, while our objective is to investigate the functional divergence among clusters.

#### 2.6.3 Trajectory and Pseudo‐Time Analysis

For the mesoderm progression dataset, we used Monocle3^[^ ^23^ ^]^ and Palantir^[^ ^24^ ^]^ to infer cell‐type trajectory and pseudo‐time, following the Google Colab tutorial from Margaret.^[^ ^25^ ^]^ We tuned Monocle3's parameter to obtain a trajectory without outlier cell types, using n_neighbors of 100 and min_dist of 0.9. We ran Palantir with default parameters in the tutorial. The minimum spanning trees of the two cell‐type trajectories were obtained by supplying the reversed adjacency matrix to Networkx's “minimum_spanning_tree” function.

For the embryogenesis dataset, we used scDHA and scTEP software to infer pseudo‐time, as they did in their original publications.^[^ ^26^, ^27^ ^]^ Both software were run with default parameters as provided in their tutorials. The cell type annotations were sorted in the developmental order, and the Pearson correlation coefficients were calculated between the ordered annotations and inferred pseudo‐time values. Furthermore, by replacing the 4‐cell label with the ordered subsets, the Pearson correlation coefficients were calculated between the updated labels and inferred pseudo‐time values.

## 3 Results

### 3.1 Ceitea Accurately Infers Clusters in Both Simulated and Real‐World Datasets Compared to State‐of‐the‐Art Clustering Tools

The clustering ability of CeiTEA was evaluated in both single‐layer simulated datasets and real‐world scRNA‐seq datasets with golden annotations. The performance of CeiTEA is benchmarked against several state‐of‐the‐art clustering tools, i.e., Louvain, Leiden, K‐means, spectral clustering, agglomerative clustering with four different linkages (i.e., single, complete, average, and ward), and SEAT. The adjusted Rand Index (ARI) and adjusted mutual information (AMI) were the primary metrics to evaluate the clustering quality of all methods.

As evident from the heatmap presented in Figure 2A, on average, CeiTEA consistently outperformed the other methods in terms of ARI and AMI scores, demonstrating its superior performance in simulated datasets. Across simulations at different levels of edge noise and among strongly and weakly defined clusters, CeiTEA also generated relatively reliable partitions compared to other methods (Figure S4A,B, Supporting Information). CeiTEA achieved high ARI and AMI scores in median (0.9933 and 0.9860, respectively) as the number of clusters and nodes increased, implying the ability to maintain high‐quality clusterings in scenarios with complex clusters. Figure 2E,F provide detailed comparisons of ARI and AMI scores, which further shows that CeiTEA outperformed other tools at different levels of edge noise and the wellness of cluster definitions. In the presence of edge noise (10%), the average performance of CeiTEA remained relatively stable with only minor declines in ARI scores (from 0.9979 to 0.9967 and from 0.9926 to 0.9638 in median for strongly and weakly defined cases, respectively) and AMI scores (from 0.9966 to 0.9921 and from 0.9778 to 0.9611 in median for strongly and weakly defined cases, respectively), exhibiting more resilience against edge noise. Regarding the wellness of cluster definitions, on the other hand, CeiTEA also showed a comparably higher average power to estimate the correct partitions and exhibited its versatility in various scenarios, with ARI and AMI of 0.9977 and 0.9954 for strongly defined cases, and 0.9883 and 0.9708 for weakly defined cases. In terms of the entropy of the partitions obtained from different tools, CeiTEA was able to generate partitions with lower entropy values on average (Figure S2C as well as Figure S4C, Supporting Information), which implies the ability to estimate partitions with minimized topological entropy.

We also evaluated CeiTEA on nine real scRNA‐seq datasets with gold standard cell type labels. We collected five datasets, namely Yan, Deng, Biase, Blakeley, and Goolam, from human or mouse embryos at different stages of development,^[^ ^28^, ^29^, ^30^, ^31^, ^32^ ^]^ and four datasets, namely Kumar, Trapnell, Kolodziejczyk, and Xin, which profile different cell types in single‐cell resolution.^[^ ^33^, ^34^, ^35^, ^36^ ^]^ In most samples, CeiTEA demonstrated reliable clustering results (Figure S5, Supporting Information). Specifically, Figure 2B provides a comprehensive comparison of CeiTEA's performance against other tools, revealing that CeiTEA consistently achieved higher ARI and AMI scores across the majority of samples. Additionally, CeiTEA generated partitions with the lowest topological entropy (Figure 2D), underscoring its capability to maintain community structures effectively. In contrast, other tools produced partitions that closely approximated the golden annotations but exhibited greater topological entropy, highlighting CeiTEA's superior ability to preserve the integrity of community structures while managing topological entropy.

In addition, we subsequently applied both CeiTEA and SEAT to the structural entropy objective to illustrate the advantages of topological entropy over structural entropy. In single‐layer simulations, topological entropy consistently outperformed structural entropy in both methods, as demonstrated in Figure S6A,B (Supporting Information). Notably, the advantage of topological entropy was significant in CeiTEA, whereas SEAT showed less pronounced improvement, highlighting the effectiveness of the partition candidate selection and ILP in CeiTEA. Similarly, in real datasets, topological entropy yielded superior partitions in six out of nine samples, as indicated by both AMI and ARI values (Figure S6C, Supporting Information).

### 3.2 CeiTEA Adaptively and Accurately Reconstructs Hierarchical Structures in Simulations Compared to the Other Hierarchical Tools

The hierarchy inference of CeiTEA was assessed across simulated multi‐layer datasets and benchmarked against traditional hierarchical clustering (HC) methods with single, complete, average, and ward linkages as well as SEAT. The clustering information distance (CID) metric was employed to assess the similarity between the true and estimated hierarchies from different tools.

**Figure 3:**
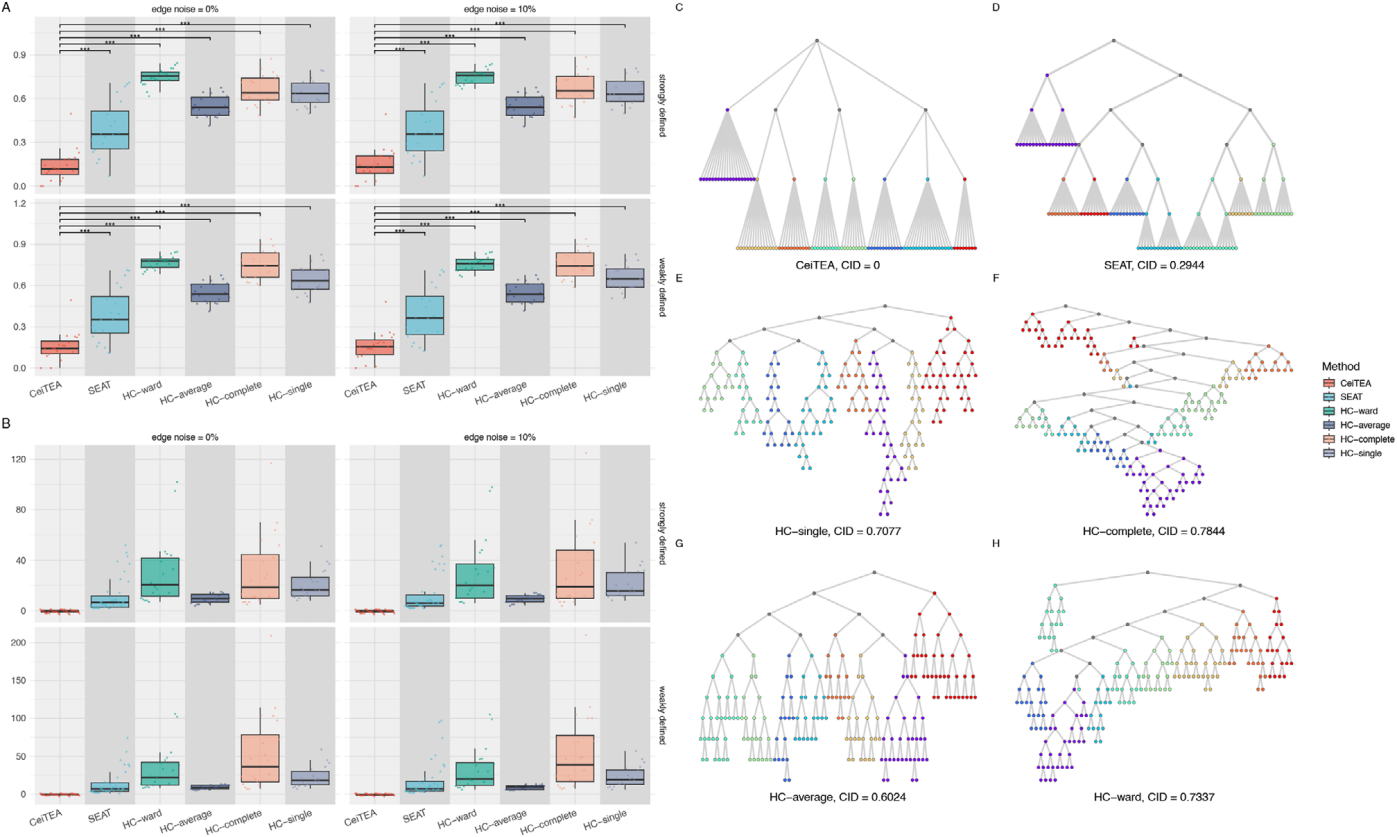
Evaluations of hierarchies estimated by CeiTEA and common hierarchical clustering tools on simulated datasets with hierarchical structures. A) Boxplots of comparisons in clustering information distances (CIDs) from the ground truths for hierarchies generated by CeiTEA, SEAT, and four HC configurations. B) Boxplots of height differences of hierarchies generated by various tools and true hierarchies. C–H) Hierarchical structures generated by CeiTEA, SEAT, and four HC configurations, under the simulation case with 100 nodes. CeiTEA retrieved exactly the same structure as the ground truth from data. The node color, except for grey, indicates that the children belong to the same partition. The CID values are marked below all hierarchies.

Overall, CeiTEA provided significantly low CIDs (0.1423 in median and 0.15 in mean) in simulated datasets, much lower compared to 0.3874 from SEAT and 0.6653 from HC methods. Specifically, **Figure** **3**A shows that such difference is statistically significant across a range of simulated datasets, thereby highlighting CeiTEA's effectiveness in extracting more accurate structures from similarity or affinity matrices, compared to SEAT and the traditional HC methods. Moreover, CeiTEA was able to reproduce the hierarchies with approximately the same heights as the true ones (Figure 3B) as well as the true hierarchical multi‐nary structures (Figure 3C–H). Taking the simulated case of 100 nodes with no edge noise and strong community definition as an example, CeiTEA generated exactly the same hierarchical structure as the ground truth with all leaf nodes of matched leaf heights (Figure 3C), which indicates the hierarchy retrieved the true relationship of parents and children and hence the subclusters were also correctly reproduced. In contrast, SEAT produced a slightly deeper hierarchy in which some internal nodes acted as parents but corresponded to none of the actual subclusters (Figure 3D). While SEAT deliberately merged leaf nodes to construct a multi‐nary hierarchy, it was unable to infer the multi‐nary structures beyond the bottom layers, resulting in a CID of 0.2944. Traditional hierarchical clustering tools only generated binary structures and extra merging steps were necessary to determine the partitions, while certain internal nodes could represent actual partitions, generating even higher CID values around 0.7071 (Figure 3E–H).

In particular, CeiTEA was able to generate hierarchies with better interpretability. Across all simulated datasets, specifically, the heights of leaf nodes of hierarchies produced by CeiTEA were more uniform and more closely approximated the heights of the true hierarchies on average (Figure S7, Supporting Information). Here, uniform leaf heights indicate a more consistent representation of community structures, which can facilitate the identification of meaningful relationships within the data, therefore implying the ability of CeiTEA to adaptively estimate underlying hierarchical structures from data.

We again applied both CeiTEA and SEAT to the structural entropy objective to compare the effectiveness of the two entropy measures regarding hierarchical structure. In multi‐layer simulations, topological entropy consistently outperformed structural entropy in both methods, achieving lower CID values, as shown in Figure S8A (Supporting Information). As before, the advantage of topological entropy was particularly pronounced in CeiTEA, while SEAT demonstrated a less significant difference, further validating the effectiveness of CeiTEA's adaptive hierarchy construction. For instance, in the previous simulation case, the CeiTEA hierarchy under the SE objective closely replicated the original structure (Figure S8B, Supporting Information). Specifically, CeiTEA achieved a CID value of 0.074, which is lower than the CID values for SEAT hierarchies under both top‐down (0.2944) and bottom‐up (0.3568) strategies, indicating the superior performance of CeiTEA in preserving hierarchical accuracy, even under the SE objective.

### 3.3 CeiTEA Hierarchy Concurs with the Differentiation Order of Cells in Mesoderm Progression

**Figure 4:**
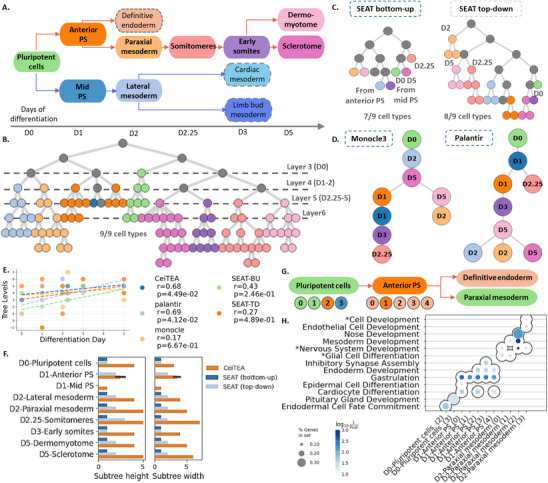
CeiTEA proposes developmental subclusters in the spatial‐temporal development process of mesoderm. A) Schematic of human mesoderm development. Nodes with dashed borders are cell types that are absent from the data. B) CeiTEA hierarchy. Gray nodes represent partitions with mixed cell types, while the remaining nodes are colored according to the specific cell types of their member cells. C) SEAT bottom‐up and top‐down hierarchies. D) Trajectory minimum spanning tree from Monocle3 and Palantir. E) Scatters and regression lines showing the correlation between the differentiation day and the corresponding tree levels from CeiTEA, Palantir, Monocle3, SEAT (bottom‐up), and SEAT (top‐down). The legend shows the correlation coefficients and *p*‐values. F) Bar plots of subtree height and width from SEAT and CeiTEA hierarchies across different cell types. G) Subclusters on Layer 6, ordered by the differentiation lineage of cell types. Node color indicates the differentiation direction revealed by GO analysis, while nodes that do not follow a specific differentiation direction retain the color of their parent cell type. H) The dot plot lists the top three GO terms enriched by the top fifty marker genes in subclusters of Plenipotent cells, Mid‐PS, and Lateral mesoderm cells in layer 6.

In this section, we validate the hierarchy constructed by CeiTEA using a scRNA‐seq dataset of cell types that adhere to a differentiation hierarchy. We utilized a scRNA‐seq atlas on human mesoderm progression from pluripotency to tissue specification.^[^ ^37^ ^]^ Through successive lineage decisions and intermediates, pluripotent cells acquire distinctive fates, including somites, sclerotome/dermomyotome, limb buds, and heart. As shown in **Figure** **4**A, pluripotent stem cells (H7hESC) on day 0 differentiate into anterior and mid primitive streaks (Anterior PS and Mid PS on day 1), initiating mesoderm patterning. On day 2, the anterior primitive streak yields paraxial mesoderm and definitive endoderm, while the mid primitive streak generates lateral mesoderm. Paraxial mesoderm segments into somitomeres (on day 2.25), forming early somites on day 3. Later on day 5, somites segregate into sclerotome ventrally, which will form bone and cartilage of the spine and ribs, and dermomyotome dorsally, which will give rise to brown fat, skeletal muscle, and dorsal dermis through differentiation and morphogenesis. On the other hand, lateral mesoderm bifurcates at around day 3, producing limb bud and cardiac mesoderm. Limb bud mesoderm supports outgrowth through structure and signaling. Cardiac mesoderm develops through morphogenesis into cardiomyocytes and other heart cell types. With this dataset, we show that the CeiTEA hierarchy can capture the differentiation capacities of cell types and that CeiTEA subclusters can suggest future differentiation directions.

CeiTEA hierarchy outperforms both SEAT's top‐down and bottom‐up approaches in representing cell‐type clusters. Based on cell type annotations, the CeiTEA hierarchy demonstrates effective cell type partitioning across various hierarchical levels (Figure 4B). CeiTEA constructed a nine‐layer hierarchy, with the top five layers primarily comprising the known cell types from the ground truth annotations. Notably, Layer 5 effectively partitions the ground truth cell types, achieving an ARI value of 0.903. Beginning with Layer 6, CeiTEA identifies four additional layers of subclusters within each cell type, offering a more nuanced developmental resolution. In contrast, the SEAT hierarchies consist of only five and seven layers, resulting in fewer subclusters for cell types (Figure 4C). Moreover, the SEAT bottom‐up hierarchy failed to identify the Mid PS and Anterior PS cell types, while the top‐down hierarchy also missed the Mid‐PS cell type.

Furthermore, the hierarchical structure inferred by CeiTEA and the topological entropy metrics are consistent with the developmental order of cell types over time. In the CeiTEA hierarchy, we observe the partitioning of day 0 pluripotent cells from Layer 3 to Layer 4, while the partitioning of subsequent cell types is not yet evident. From Layer 4 to Layer 5, partitions for day 1 to day 2 cell types emerge, with the exception of the Mid PS cell type on day 1, which is the smallest cell type in the atlas. Finally, the transition from Layer 5 to Layer 6 illustrates the partitioning of cell types from day 2.25 to day 5. This hierarchical order reflects increasing cell type specialization, as early lineage bifurcations result in subtypes with greater divergences, indicating broader early potential. In contrast, later fate decisions occur under narrower constraints, demonstrating heightened specialization. The topological entropy metric in CeiTEA reflects changes in cell type specialization, with Layer 5 exhibiting a lower topological entropy of –2.48 compared to –0.93 at the bottom layer. This lower entropy value at the middle layer substantiates a clearer separation between subtypes of less differentiated early cell populations. In contrast, the finer‐grained subclusters at the bottom layer show a relatively larger topological entropy value, reflecting diminished distinctions among subpopulations of already specified later cell types. Together, the CeiTEA‐derived hierarchical structure and the topological entropy metrics are in concordance with increased developmental restriction over time elucidated by the hierarchical organization. In contrast, the SEAT hierarchies do not adhere to this developmental order. In the SEAT bottom‐up hierarchy, day 0 pluripotent cells are placed at the same layer as day 5 sclerotome cells, both situated below the day 2.25 somitomere cells. Similarly, in the SEAT top‐down hierarchy, day 0 pluripotent cells are positioned below the day 2, day 2.25, and day 5 cell types. The misalignment in SEAT hierarchies indicates a misrepresentation of the temporal progression of cell type differentiation.

For additional benchmarking, we applied Monocle3 and Palantir to reconstruct cell‐type trajectories (Figure S9A,B, Supporting Information) and assess their alignment with developmental order, highlighting CeiTEA's unique ability to integrate temporal dynamics and cell‐type specialization. The minimum spanning trees from both trajectories, rooted at day 0 pluripotent cells, are shown in Figure 4D. The Palantir trajectory tree demonstrated strong alignment with the developmental progression. We benchmarked CeiTEA against SEAT and the trajectory methods by assessing the correlation between differentiation days and node levels in the trees (Figure 4E). CeiTEA achieved a high correlation (*r* = 0.68, *p* = 0.04), comparable to Palantir (*r* = 0.69, *p* = 0.04) and outperforming SEAT (*r* = 0.43). Furthermore, we compared CeiTEA with pseudo‐time values from Monocle3 and Palantir (Figure S9C, Supporting Information). CeiTEA exhibited the strongest correlation with differentiation days, surpassing Monocle3 (*r* = 0.56) and Palantir (*r* = 0.29). Benchmarking against SEAT, Monocle3, and Palantir demonstrates CeiTEA's superior ability to integrate temporal progression and cell type specialization into a single framework, offering deeper insights into differentiation dynamics and developmental trajectories.

In addition to cell type identification, the hierarchical partition of cell‐type nodes in the CeiTEA hierarchy tree is more meaningful compared with SEAT. For each cell type, we consider its downward partitions as a subtree and extract the corresponding height and width (Figure 4F). For the SEAT bottom‐up hierarchy, no cell‐type partitions are generated. For the SEAT top‐down hierarchy, the subtree heights and widths are rather invariant across cell types, with variances of 0.4107 and 0.2143, respectively. Conversely, subtree heights and widths from the CeiTEA hierarchy vary among cell types, with much higher variances of 1.7889 and 2.8444, respectively. In particular, the somitomeres, characterized as transitional lineage intermediates between pluripotent cells and specialized structures, exhibit the largest subtree height of 5 and width of 6. This aligns with their transient nature, where rapid changes in gene expression in response to developmental signals lead to a diverse array of cell states.

Furthermore, CeiTEA subclusters can suggest future differentiation directions. We examine the biological relevance of subclusters on Layer 6 by applying gene ontology (GO) analysis on their marker genes (Table S1, Supporting Information). Regarding cell types in the early stage, there are three subclusters for the pluripotent cells, five for the Anterior PS cells, and four for the paraxial mesoderm cells (Figure 4G). Pluripotent cells are expected to differentiate into Anterior PS and Mid PS cells. Subcluster 2 of the pluripotent cells shows significant enrichment for endodermal cell fate commitment (GO:0001711, adjusted *p* = 0.0178), aligning with the Anterior PS differentiation pathway (Figure 4H). In contrast, subcluster 3 is significantly enriched for pituitary gland development (GO:0021983, adjusted *p* = 0.0459), reflecting the direction toward Mid PS. Moving to the Anterior PS, all subclusters except for subcluster 1, enrich GO terms related to endoderm, agreeing with the documented developmental direction. For the paraxial mesoderm cells, the subclusters exhibit significant enrichment for GO terms that are more specific in cell differentiation and tissue development. Notably, the GO terms are rather similar among subclusters from early cell types, while subclusters of later cell types present larger functional divergence (Figure S9D, Supporting Information). Here, subcluster 0 and subcluster 1 of early somites correspond to the differentiation direction leading to sclerotome and dermomyotome cells, respectively. Specifically, GO results suggest that subcluster 0 is related to the regulation of mesenchymal cells, highlighting its role in processes such as epithelial‐to‐mesenchymal transition and subsequent differentiation into skeletal components.^[^ ^38^ ^]^ In contrast, subcluster 1, representing the dermomyotome, is significantly enriched for skeletal‐related terms, as the dermomyotome serves as a source of myogenic progenitor cells that migrate into the myotome to form skeletal muscle.^[^ ^39^ ^]^

### 3.4 CeiTEA Reveals Embryogenesis Subclusters with Different Developmental Potentials

**Figure 5:**
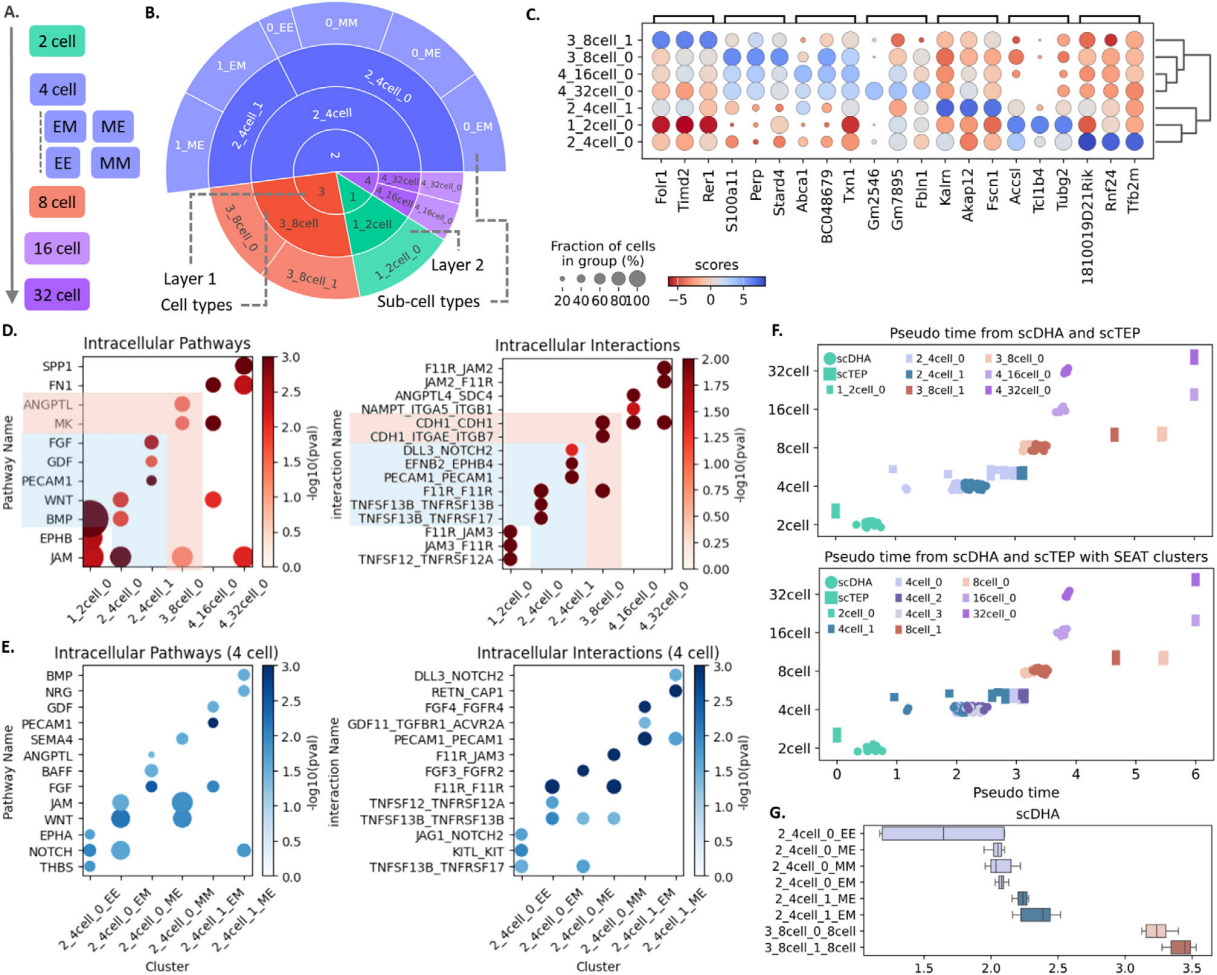
CeiTEA suggests communication subclusters in early mouse embryo developmental cells. A) Classification of early mouse embryo developmental cells. B) Sunburst plot of two‐layer CeiTEA clustering results and cell type annotations. C) Top three marker genes of clusters in low‐level subclusters of CeiTEA. D) Top three pathways and ligand‐receptor interactions in Layer 1 of CeiTEA. E) Top three pathways and ligand‐receptor interactions in two 4‐cell subclusters in Layer 1 of CeiTEA. F) Pseudo‐time from scDHA (circle) and scTEP (square) on different cell types, colored by CeiTEA Layer 2 labels (top) and SEAT clusters in the bottom layer (bottom). G) Boxplot of pseudo‐time distributions in 4‐cell and 8‐cell subsets from scDHA. The labels and their representative cell types are: meridional (ME), equatorial (EM), double equatorial (EE), and double meridional (MM).

We obtained scRNA‐seq data from different stages of early mouse embryo development^[^ ^32^ ^]^ to test the subclustering abilities of CeiTEA. The data contained transcriptomes of individual cells at the 2‐cell, 4‐cell, 8‐cell, 16‐cell, and 32‐cell stages (**Figure** **5**A). Notably, 4‐cell embryos were further classified into four subclasses based on the division patterns relative to the position of the second polar body: meridional (M) and equatorial (E), where the combinations resulted in the groups ME, EM, EE, and MM. This classification provides informative grouping to accurately evaluate CeiTEA's performance in extracting clusters and subclusters with distinct developmental characteristics.

CeiTEA generated a two‐layer hierarchy (Figure 5B) that accurately predicted cell types and adhered to developmental order. In Layer 1 (top layer), CeiTEA identified four major clusters, yielding an ARI of 0.981 compared to the ground truth, indicating a high degree of agreement. On the other hand, the heights of major clusters in SEAT vary, suggesting meaningless heights of internal nodes in binary tree modes (Figure S10, Supporting Information). We observed that the 32‐cell and 16‐cell clusters are merged, the same as in all SEAT results. The expression similarities between the 16‐cell and 32‐cell stages are reflected in the absence of distinct marker genes, as shown in Figure 5C. In the subsequent Layer 2 (bottom layer), CeiTEA identified two subclusters for both the 8‐cell and 4‐cell stages. Furthermore, the topological entropy metric employed by CeiTEA suggests a larger divergence among major cell types (Layer 1 topological entropy at ‐1.030), while the level of divergence is smaller for subclusters (Layer 2 topological entropy at ‐0.866). For SEAT, only the top‐down strategy identified subclusters for both stages. Furthermore, SEAT generated four 4‐cell subclusters, while such partitions are considered entropy‐increasing, generating higher‐than‐zero TE values (average TE value = 0.0036) with respect to their parent nodes.

In analyzing the differences between the two subsets of 8‐cell mouse embryos based on marker genes (Figure 5C), associated biological processes, and signal pathways (Figure 5D), we can observe distinct functional characteristics that reflect their developmental roles. The subset 3_8cell_0 is characterized by genes implicated in stress responses and cellular protection mechanisms. Notably, the presence of marker gene *S100a11* indicates a role in calcium binding and maintaining cellular homeostasis under stress. This subset's cellular interactions primarily involve JAM, MK, and ANGPTL signaling pathways, which facilitate cell adhesion, proliferation, and metabolic regulation. Specifically, *F11R*, *CDH1*, and integrins enhancing adhesion and migration are present. In contrast, subset 3_8cell_1 emphasizes metabolic processes, prominently featuring the marker gene *Folr1*, which is involved in folate transport. While no significant interactions are identified, GO analysis further supports that this subset is actively engaged in apoptosis regulation and metabolic processes related to mitochondria and lipids (Table S2, Supporting Information).

Moreover, the two 4‐cell subsets, with different composition of division patterns, could represent diverse developmental potentials. Previous research suggests that the ME and EM patterns are associated with higher probabilities of successful development due to their balanced inheritance of cellular materials, while MM and EE patterns exhibit more variability and reduced viability. Within 4‐cell, CeiTEA generated two subsets: the subset 2_4cell_0 consists of EE and MM cells, as well as a portion of the ME and EM cells, while the subset 2_4cell_1 consists of only ME and EM cells. In accordance with the previous research, the difference in intra‐signaling pathways (Figure 5D) between these two subsets revealed that 2_4cell_1 containing ME and EM cells already demonstrated specialized functions reflecting developmental directions during early embryogenesis. Specifically, 2_4cell_1's PECAM1, GDF, and FGF pathways suggest a specialization toward vascularization and tissue formation. Furthermore, the 2_4cell_1 marker interaction *EFNB2_EPHB4* is essential for guiding cell migration and positioning during angiogenesis, while *PECAM1*‐related interaction is crucial for maintaining endothelial integrity and facilitating cell‐cell adhesion during angiogenesis.^[^ ^40^, ^41^ ^]^ In contrast, the presence of both WNT and BMP pathways in 2_4cell_0 indicates a less specific differentiation direction. Intra‐signaling pathways among pattern subsets (Figure 5E) further validate the higher probability for successful embryogenesis for the ME and EM cells in 2_4cell_1, as they present pathways related to the critical formation of functional inner cell mass (ICM). Specifically, the NRG signaling pathway enriched in the ME subset within 2_4cell_1 is believed to play a role in the communication between cells during early development, influencing the fate of pluripotent stem cells within the ICM. Similarly, the GDF signaling pathway in the EM subset within 2_4cell_1 promotes the proliferation and functionality of trophectoderm cells, which are critical for proper ICM development.

Cellular pseudo‐time further validates the difference in CeiTEA‐proposed subsets for 4‐cell and 8‐cell. We collect pseudo‐time results from scDHA and scTEP,^[^ ^26^, ^27^ ^]^ whose Pearson correlation coefficients with the developmental order are 0.9186 and 0.9170, respectively. Examining the pseudo‐time results with respect to the CeiTEA clusters in Layer 2 revealed a clear separation between subsets of 4‐cell and 8‐cell in pseudo‐time (Figure 5F top). Such separation between 4‐cell subsets is further validated by the elevation in Pearson correlation coefficients for both methods when enforcing the developing order placing 2_4cell_0 before 2_4cell_1, reaching 0.9393 and 0.9297 for scDHA and scTEP, respectively. On the other hand, the two methods generated pseudo‐time for 8‐cell subsets that were well‐separated but conversely ordered, as shown in Figure 5F. Conversely, SEAT suggested four 4‐cell subsets that showed less clear partition in pseudo‐time values (Figure 5F bottom).

Further investigation into the pseudo‐time results from patterns within 4‐cell subsets (Figure 5G and Figure S11A, Supporting Information) agreed with our previous observations based on cellular interactions and known developmental potentials. Specifically, EE and MM present smaller pseudo‐time compared to EM and ME, in agreement with their smaller developmental potential. EM and ME from 2_4cell_0 also present smaller pseudo‐time compared to their counterparts from 2_4cell_1, in accordance with their less specified state as revealed by pathway analyses. Nonetheless, the pseudo‐time distribution on SEAT 4‐cell subsets does not consistently align with known developmental potentials of division patterns (Figure S11B, Supporting Information).

### 3.5 CeiTEA Reveals Tumor‐TME Boundaries on Spatial Transcriptome Dataset

In this section, we aim to investigate the efficacy of CeiTEA hierarchy clustering in retrieving hierarchical spatial regions from spatial transcriptome (ST) data. Furthermore, we wish to examine the possible biological insights revealed by CeiTEA hierarchical clusters, for which we considered two datasets from cancerous tissues: a HER2‐positive breast cancer (BC) dataset comprising eight samples^[^ ^42^ ^]^ and a pancreatic ductal adenocarcinoma (PDAC) dataset consisting of one sample.^[^ ^18^ ^]^ Here, we demonstrate the robustness of CeiTEA in delineating tumor and tumor microenvironment (TME) regions, identifying subregions at the tumor‐TME boundary, and characterizing heterogeneity within both tumor and TME regions. We also compare its performance with two widely used ST clustering methods, Louvain and Leiden.

CeiTEA constructed a two‐layer hierarchical structure for each sample in both datasets, with the exception of one sample from the BC dataset, which exhibited a single‐layer hierarchy (Figure S12, Supporting Information). The hierarchical structure effectively captures the organization of tumor and TME regions in Layer 1 and finer cell type distinctions in Layer 2. In particular, we observed larger breadth of tumor clusters in Layer 1 compared to the TME clusters (Figure S13A, Supporting Information), reflecting the greater heterogeneity and diversity of cell states within the tumor region compared to the TME. Topological entropy metrics further demonstrate CeiTEA's ability to elucidate divergence levels between these layers. Specifically, agreeing with our observation above, tumor‐TME‐like clusters in Layer 1 exhibit lower divergence, with an average topological entropy of –0.770, reflecting distinct and well‐separated clusters. In contrast, Layer 2 clusters, representing annotated cell types, reveal greater divergence, as indicated by an average topological entropy of –1.219 (Figure S13B, Supporting Information).

**Figure 6:**
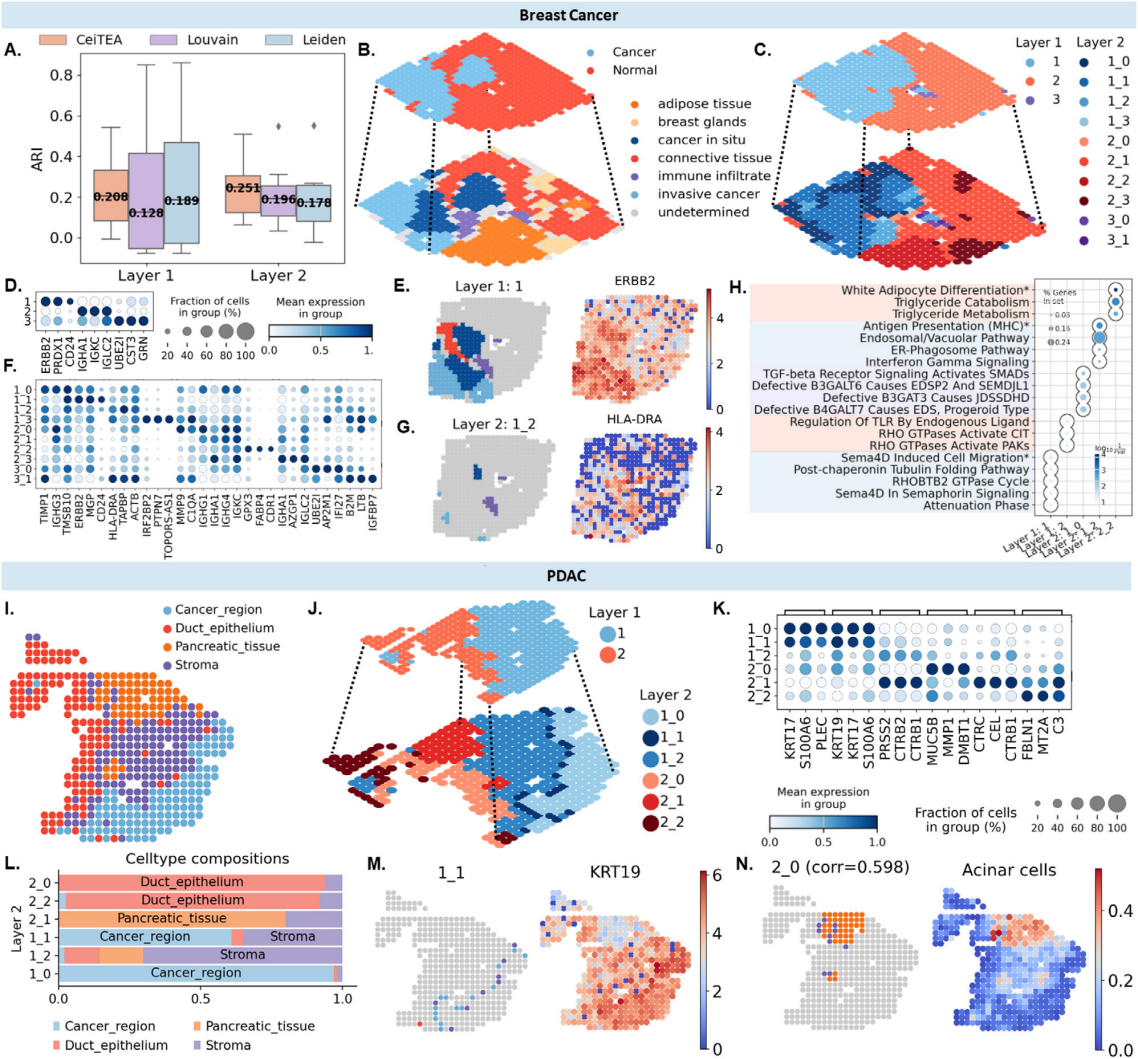
CeiTEA reveals tumor‐TME boundaries on breast cancer and PDAC ST datasets. A) Boxplot of ARI values for CeiTEA, Louvain, and Leiden results across samples on both datasets. B–H) CeiTEA results on the breast cancer sample H1. B) Layered annotations of the breast cancer sample H1. C) Clustering result of CeiTEA. D) Top three marker genes of clusters in Layer 1 (top layer). E) Cluster 1 of Layer 1 and its marker genes *ERBB2*. F) Top three marker genes of clusters in Layer 2 (bottom layer). G) Cluster 1_2 of Layer 2 and its marker genes *HLA‐DRA*. H) The dot plot lists the top five GO terms enriched by the marker genes in clusters 1 and 2 from Layer 1 and clusters 1_0, 1_2, and 2_2 from Layer 2. I–N) CeiTEA results on the PDAC sample. I) Regional annotation of the PDAC sample. J. Clustering result of CeiTEA. K. Top three marker genes of clusters in Layer 2. L. Cell type compositions in Layer 2 clusters. Clusters having a cell type with a majority of over 30% are displayed. M. Left: Spatial distribution and contained cell types in cluster 1_2 of Layer 2; right: marker gene *KRT19* of cluster 1_2. N. Left: Spatial distribution and contained cell types in cluster 2_0 of Layer 2; right: deconvolution results of acinar cells. ARI: adjusted rand index.

CeiTEA exhibits robust clustering performance compared to common clustering methods, including Louvain and Leiden, across multiple samples in two datasets. The clustering performance of Layer 1 was evaluated using ARI against annotated tumor and TME regions, while Layer 2 was assessed against cell type annotations. Results indicate that CeiTEA outperforms both methods, as demonstrated by ARI in **Figure** **6**A. Specifically, Layer 1 clustering achieved a median ARI of 0.208, surpassing Louvain's 0.128 and Leiden's 0.189. Similarly, Layer 2 exhibited higher consistency with annotated cell types, yielding median ARIs of 0.251, compared to 0.196 for Louvain and 0.178 for Leiden. Clustering results are visualized on spatial transcriptomics slices in Figure S14 (Supporting Information).

To assess the biological validity of the clustering results, we analyzed sample H1, which exhibited the highest ARI values for both layers (0.64 for Layer 1 and 0.36 for Layer 2). Based on the original spot annotations (Figure 6B), the top layer of CeiTEA's clustering results effectively delineated tumor and TME regions (Figure 6C). The marker genes identified in Layer 1 clusters substantiate the biological relevance of these tumor‐TME‐like clusters (Figure 6D). Notably, *ERBB2*, a well‐known marker gene for breast cancer, was identified as a key marker for the tumor‐representing cluster 1 in the top layer (Figure 6E). In the tumor region, Layer 2 clusters revealed distinct tumor‐TME boundary clusters (Figure S15, Supporting Information). The marker genes associated with Layer 2 indicate the presence of immune‐related markers in these boundary clusters (Figure 6F). For instance, boundary cluster 1_2, which comprises immune infiltrates as well as both in situ and invasive cancer, is characterized by the immune gene *HLA‐DRA* (Figure 6G).

GO analyses of the marker genes provide additional functional validation for the clusters identified by CeiTEA (Figure 6H; Table S3, Supporting Information). Consistent with our previous findings, the marker genes of the tumor‐representing cluster 1 are enriched in Sema4D‐related pathways, which are associated with breast cancer metastasis.^[^ ^43^ ^]^ Similarly, the marker genes of the TME‐representing cluster 2 are enriched in immune‐related RHO GTPase GO terms, which have recently been shown to have tumor suppressor functions.^[^ ^44^ ^]^ Furthermore, the adipose tissue‐representing cluster 2_2 is enriched in a GO term associated with adipocyte differentiation (R‐HSA‐381 340, adjusted *p* ⩽ 0.0001). The Layer 2 boundary clusters within tcluster 1 exhibit distinct functions related to tumor‐TME interactions. Specifically, cluster 1_0, which comprises connective tissue and invasive cancer, is enriched for the GO term associated with SMAD activation (R‐HSA‐2173789, adjusted *p* = 0.0011), a process known to be involved in epithelial plasticity, tumor‐stroma interactions, invasion, and metastasis in breast cancer.^[^ ^45^ ^]^ Conversely, cluster 1_2, which includes immune infiltrates and two cancer types, shows enrichment for a GO term related to major histocompatibility complex antigen presentation (R‐HSA‐983170, adjusted *p* ⩽ 0.0001), highlighting a response to tumor infiltration at the tumor‐TME boundary.

In the PDAC dataset (Figure 6I), CeiTEA generates a hierarchical clustering that aligns with cell type annotations, achieving an ARI of 0.508 (Figure 6J). As with previous analyses, we validate the clusters by examining their marker genes (Figure 6K). For instance, the tumor subcluster 1_0 in Layer 2 is characterized by the marker gene *KRT17*, which is a recognized marker for PDAC. Similarly, the *CTRC* gene, marking the pancreatic tissue‐representing cluster 2_1 in Layer 2, encodes chymotrypsin C, produced by pancreatic acinar cells. Upon comparing the cell type annotations with the clustering results in the bottom layer, we observe clusters containing mixed cell types, as well as distinct subclusters within specific cell types (Figure 6L). For example, cluster 1_1 serves as a tumor‐TME boundary cluster, marked by the PDAC marker gene *KRI19* (Figure 6M). The duct epithelium region is primarily partitioned by clusters 2_0 and 2_2 (Figure S16A, Supporting Information). Notably, subcluster 2_2 is marked by *FBLN1*, which is associated with the epithelial‐to‐mesenchymal transition (EMT) during cancer progression.^[^ ^46^ ^]^ Thus, subcluster 2_2 may represent an EMT region within the duct epithelium.

Cell‐type deconvolution results for the PDAC dataset further validate the Layer 2 clusters. We obtained cell‐type deconvolution results from SPOTlight^[^ ^47^ ^]^ and examined their correlation with Layer 2 clusters, as well as with Louvain and Leiden labels (Figure S16B,C, Supporting Information). Across all cell types, CeiTEA Layer 2 clusters exhibit higher maximum absolute correlation coefficients compared to Louvain and Leiden, with average |*r*| values of 0.337, 0.317, and 0.328, respectively (Figure S16D, Supporting Information). For instance, pancreatic tissue cluster 2_0, marked by acinar cell genes, correlates with acinar cells at a Pearson *r* of 0.60, whereas the highest correlations for Leiden and Louvain labels are 0.57 and 0.46, respectively. Between the clusters 2_0 and 2_2 that partition the duct epithelium region, cluster 2_0 demonstrates the highest correlation with ductal cells (Pearson *r* = 0.69), while Leiden and Louvain labels achieve lower correlations (highest *r* of 0.58 and 0.67, respectively). Additionally, CeiTEA achieves correlations with eight cell types (|*r*| ⩾ 0.3), matching that of Leiden and surpassing Louvain (Figure S16E, Supporting Information). Notably, Louvain clusters fail to correlate with endothelial cells, achieving a maximum |*r*| of 0.21, while CeiTEA cluster 2_2 shows a significantly higher correlation (highest |*r*| = 0.41 compared to Louvain's 0.33).

## 4 Discussion

In the era of single‐cell omics, understanding the hierarchical relationships between various cell types and subtypes is crucial to understanding the intricate organization of biological systems. However, the challenge lies in accurately representing these relationships, as traditional structures often fail to capture the adaptive nature of biological hierarchies. CeiTEA addresses this problem by employing an adaptive hierarchy that allows for the construction of multi‐nary trees without rigid constraints. By introducing a novel measure termed topological entropy (TE), CeiTEA facilitates the creation of a minimal TE hierarchy, resulting in a rooted, unbalanced multi‐nary tree that optimally represents the relationships and diversity among cell types and subtypes. By minimizing TE through eigen‐decomposition and linear programming, CeiTEA facilitates a more accurate representation of cellular diversity while preserving the depth and breadth of cellular relationships.

The CeiTEA method introduced in this work offers several key advantages over existing approaches to analyze the hierarchical structure of biological data. Crucially, CeiTEA abandons the rigid constraints imposed by binary or balanced tree models that can fail to capture the inherent complexity and adaptivity of biological hierarchies. With the novel TE measure that incorporates both internal cohesion and external relationships, CeiTEA is able to construct an adaptive hierarchy that more faithfully represents the depth and breadth of cell‐type relationships. This is in contrast to methods like SEAT that rely solely on structural entropy and binary tree structures. The evaluation of CeiTEA on simulated multi‐layer datasets further demonstrated its superior performance in retrieving hierarchical structures. In particular, CeiTEA showed a statistically more consistent and accurate reconstruction of hierarchies compared to SEAT, even in the presence of confounding factors such as cluster ambiguity and edge noise. By not imposing rigid structural constraints, CeiTEA was able to better capture the nuanced relationships between cell types and subtypes. In general, the adaptive and information‐theoretic foundation of CeiTEA represents an important advancement in the analysis of complex biological hierarchies beyond what is possible with existing clustering‐based methods.

The hierarchical structure inferred by CeiTEA and the associated topological entropy metrics can provide valuable insights into the developmental potency of cells. The CeiTEA hierarchy effectively captured the differentiation order of cell types in the human mesoderm progression dataset, aligning with the known developmental sequence from pluripotency to tissue specification. The partitioning of cell types across the hierarchical layers corresponded to increasing cellular specialization over time. Furthermore, early lineage bifurcations resulted in subtypes with broader developmental potential, as reflected in the higher topological entropy values at the lower layers of the hierarchy. In contrast, later fate decisions occurred under narrower constraints, leading to more specialized cell types with lower topological entropy. Similarly, in the mouse embryogenesis datasets, the first layer represents major cell types, with a low TE value suggesting a larger divergence, while the second layer consists of subclusters, with a slightly higher TE value representing a relatively smaller divergence. Overall, the CeiTEA hierarchical structure and TE metric provide a principled way to infer cellular divergence that reflects the relative developmental potency of different cell populations or inner dynamics within cell types, offering a powerful framework for analyzing and interpreting single‐cell transcriptomic data in the context of tissue specification and developmental biology.

The identification of biologically meaningful subsets within the CeiTEA hierarchy underscores the method's capacity to reveal functional and developmental distinctions among cell types. For instance, in the analysis of human mesoderm development, subclusters derived from pluripotent cells demonstrated significant enrichment for specific GO terms, such as endodermal cell fate commitment, reflecting their anticipated differentiation directions. Similarly, distinct marker genes in the early somite subclusters indicated their roles in sclerotome and dermomyotome differentiation, highlighting the functional divergence in later developmental stages. In the analysis of mouse embryo 4‐cells, the two subsets generated differed significantly in their composition of cell patterns and developmental potentials. Only one subset, which comprised primarily of ME and EM pattern cells, demonstrated specialized functions related to vascularization and tissue formation, as indicated by the enrichment of pathways such as PECAM1 and GDF. This distinction is consistent with previous research suggesting that ME and EM patterns are associated with a higher probability of successful development due to their balanced inheritance of cellular materials. Further supporting these findings, pseudo‐time analysis showed a clear separation between the two subsets, with the above subset exhibiting a higher likelihood of progressing toward functional inner cell mass formation. Additionally, in the breast cancer dataset, CeiTEA identified distinct tumor subregions and their microenvironment, partitioning them into clusters that reflected the tumor, TME, and tumor‐TME boundaries. This ability to distinguish between these contexts is crucial for understanding tumor heterogeneity. The corresponding scRNA‐seq cell‐type deconvolution results on the PDAC dataset corroborate these findings, showing that CeiTEA clusters align well with both ST and scRNA‐seq data in the tumor microenvironment. This capability of CeiTEA to elucidate such nuanced divergence in interactions, developmental potentials, and tumor heterogeneity enhances our understanding of cellular differentiation and progression, particularly in complex contexts such as embryogenesis and cancer.

The dependency on the parameter β and the number *n*_*e*_ of involved eigenvectors is a notable limitation of the CeiTEA algorithm, as it significantly impacts the quality of the resulting partitions. Since β influences the matrix used for eigendecomposition and *n*_*e*_ affects the number of potential candidates, selecting inappropriate values could lead to suboptimal partitions and increased topological entropy, thereby compromising the algorithm's effectiveness. To address this limitation, CeiTEA employs a systematic approach that tests a predetermined range of β values. By evaluating multiple settings, the algorithm can identify a set of eigenvectors that best capture the graph's structure. Additionally, CeiTEA limits the focus to a fixed number of eigenvectors that yield the lowest entropy values, which helps manage computational complexity while ensuring that meaningful partitions are prioritized. This method enhances the robustness of the algorithm against variations in β and *n*_*e*_, allowing it to achieve more reliable results despite its inherent dependency on this parameter.

Like the Louvain and Leiden algorithms, CeiTEA has the limitation of not being able to generate a predefined number of clusters. While these established methods effectively identify community structures through modularity optimization, they do not allow users to specify an exact cluster count. However, as a hierarchical clustering method, CeiTEA allows users to select a specific layer that approximates the desired number of clusters. Furthermore, the parameters in CeiTEA function similarly to the resolution parameter in these community detection algorithms, although tuning neither parameter guarantees that the resulting clusters will meet user‐defined criteria. To overcome the limitation of parameters, the algorithm may benefit from post‐processing techniques or integration with visualization methods. Such approaches can help users identify optimal parameter configurations that align with their analytical objectives, ultimately enhancing the utility of CeiTEA in generating meaningful clusters.^[^ ^28^, ^29^, ^30^, ^31^, ^32^, ^33^, ^34^, ^35^, ^36^ ^]^

## Conflict of Interest

The authors declare no conflict of interest.

## Author Contributions

B.‐T. and S.‐L. contributed equally to this work. S.C.L. designed methodology, provided support for projects and funds, and performed project supervision and validation. B.T. performed software implementation and conducted experiments. B.T. and S.Y.L. analyzed the results. B.T. and S.Y.L. performed document writing–original draft, review, and editing. S.C.L and M.W. reviewed the manuscript. All authors have read and agreed to the published version of the manuscript.

## Supporting information

Supporting Information

## Data Availability

The affinity/similarity matrices of nine scRNA‐seq samples involved in this study are collected from the preprocessing in SEAT. The scRNA‐seq datasets originated from: Blakeley, Biase, Yan, Goolam, Kolodziejczyk, Trapnell, Kumar, Deng and Xin.
