# Supplementary material for: CeiTEA: Adaptive Hierarchy of Single Cells with Topological Entropy
Source: Adv Sci (Weinh). 2025 Apr 17;12(26):2503539. doi: 10.1002/advs.202503539 (PMC12245133; doi:10.1002/advs.202503539)
Supplement: Supplementary file 1 — Supporting Information [file ADVS-12-2503539-s001.pdf]

ADVANCED SCIENCE
Open Access

Supporting Information

for *Adv. Sci.*, DOI 10.1002/advs.202503539

CeiTEA: Adaptive Hierarchy of Single Cells with Topological Entropy

*Bowen Tan, Shiying Li, Mengbo Wang and Shuai Cheng Li**

Supplymentary Materials

March 25, 2025

Contents

1 Supplementary Methods **3**
- 1.1 Derivation of the objective . . . 3
- 1.2 Approximate solution and candidate selection . . . 4
- 1.3 Greedy implementation . . . 5
- 1.4 Construction of a stratified hierarchy . . . 5
- 1.5 Estimation of the β range . . . 5

2 Supplementary Tables **7**

3 Supplementary Figures **9**

List of Tables

1 Top three GO terms results from Layer 5 clusters in the Koh dataset. . . . 7
2 GO results from Layer 2 clusters in the Goolam dataset. . . . 8
3 Top five GO terms from clusters in the breast cancer H1 sample. . . . 8

List of Figures

1 CeiTEA candidate generation . . . 9
2 Resources used by CeiTEA. . . . 10
3 The minimum topological entropy of partitions by eigenvectors, maximum adjusted Rand Index of partitions by eigenvectors and maximum correlation coefficient between the truth dummy labels and eigenvectors with each β value in the estimated range. The red dashed vertical line indicates the corresponding β where the minimum or maximum value is obtained. 11
4 Heatmap of ARI, AMI and topological entropy of all methods in simualted datasets across different levels of edge noise and wellness of cluster definitions. **A.** AMI scores across noise and cluster definitions. **B.** ARI scores across noise and cluster definitions. **C.** Topological entropy across noise and cluster definitions. Values surrounded by boxes indicate the maximum (for ARI and AMI) and minimum (for entropy) among all methods. . . . 12
5 Clustering results and the sorted affinity matrices of CeiTEA on real single-cell datasets. . . . 13
6 Comparisons between SE and TE of CeiTEA and SEAT on single-layer simulation and real datasets. . . . 14
7 An overview of heights of leaf nodes in hierarchies estimated by CeiTEA and other tools. The height of the true hierarchy is depicted as a black vertical line in each row. Row labels in the left are "node size/expected height when simulation/true height after simulation". . . . 15
8 Comparisons between SE and TE of CeiTEA and SEAT on simulated multi-layer datasets, and the hierarchical trees based on SE. . . . 16
9 Additional results for mesoderm dataset. . . . 17
10 Hierarchies of CeiTEA and SEAT. . . . 18
11 Pseudo-time analysis on CeiTEA and SEAT results. . . . 19
12 Overview of CeiTEA hierarchies on spatial transcriptome samples. . . . 20
13 Breadth distribution and topological entropy values of CeiTEA hierarchies on spatial transcriptome samples. . . . 21

14 Overview of clustering results on spatial transcriptome samples. 22
15 Cell type compositions in Layer 2 clusters from the BC dataset. 23
16 Additional results on the PDAC dataset. 24

1 Supplementary Methods

1.1 Derivation of the objective

Given a graph $G = (V, E; \mathbf{X})$ associated with a encoding tree $\mathcal{T}$, the topological entropy (TE) of $\mathcal{T}$ introduced in this study is defined as

$$\mathcal{K}_{\mathcal{T}}(G) = \sum_{\mu \in \mathcal{T}, \mu \neq r(\mathcal{T})} \mathcal{K}_{\mathcal{T}}(G; \mu) = \sum_{\mu \in \mathcal{T}, \mu \neq r(\mathcal{T})} -\frac{s(\mu)}{\mathrm{vol}(p(\mu))} \log_2 \frac{\frac{s(\mu)}{\mathrm{vol}(p(\mu))}}{\left(\frac{\mathrm{vol}(\mu)}{\mathrm{vol}(p(\mu))}\right)^2} \tag{1}$$

where $p(\mu)$ is the parent node of μ, $\mathrm{vol}(\mu) = \sum_{u \in V_\mu, v \in V} x_{u,v}$ is the volume of μ, $g(\mu) = \sum_{u \in V_\mu, v \notin V_\mu} x_{u,v}$ is the egress or outgoing connectivity of μ and $s(\mu) = \mathrm{vol}(\mu) - g(\mu)$ is the cohesion of μ. Generally, an encoding tree of a graph with a lower topological entropy value implies a more ordered (with less uncertainty) partitioning and a more structured hierarchy, similar to structural entropy (SE). Therefore, minimizing TE allows for a reasonable partitioning of vertices in the context of graph clustering and community detection.

Now, we focus on a plain partitioning of the whole vertex set V; that is, our aim is to partition (or cluster) V of size n into K disjoint subsets $\mathcal{P} = (P_1, \ldots, P_K)$. This is equivalent to finding a three-layer encoding tree $\mathcal{T}^{(3)}$ of G, where the nodes in the middle layer (i.e., the children of $r\left(\mathcal{T}^{(3)}\right)$) represent the partitioning $\mathcal{P}$ and each child encodes one partition P_k. Let ψ_k be the node encoding P_k and μ be the leaf node. Since $p(\psi_k) = r\left(\mathcal{T}^{(3)}\right)$, $\mathrm{vol}(p(\psi_u)) = \mathrm{vol}(G)$. Moreover, for a leaf node μ belonging to P_k, we have $p(\mu) = \psi_k$ and hence $\mathrm{vol}(\mu) = \mathrm{vol}(\psi_k)$ as well as $\mathrm{vol}(\mu) = g(\mu)$ implying $s(\mu) = 0$, which results in $\mathcal{K}_{\mathcal{T}^{(3)}}(G; \mu) = 0$. By Equation 1, thus, we have the TE of $\mathcal{T}^{(3)}$ associated with $\mathcal{P}$ as

$$\mathcal{K}_{\mathcal{T}^{(3)}}(G, \mathcal{P}) = -\sum_k \frac{s(\psi_k)}{\mathrm{vol}(G)} \log_2 \frac{s(\psi_k)}{\mathrm{vol}(G)} + \sum_k 2\frac{s(\psi_k)}{\mathrm{vol}(G)} \log_2 \frac{\mathrm{vol}(\psi_k)}{\mathrm{vol}(G)} \tag{2}$$

and the objective to find the optimal $\mathcal{T}^{(3)}_{\mathrm{opt}}$ minimizing TE is

$$\mathcal{T}^{(3)}_{\mathrm{opt}} = \arg \min_{\mathcal{T}^{(3)}, \mathcal{P}} \sum_k 2\frac{s(\psi_k)}{\mathrm{vol}(G)} \log_2 \frac{\mathrm{vol}(\psi_k)}{\mathrm{vol}(G)} - \sum_k \frac{s(\psi_k)}{\mathrm{vol}(G)} \log_2 \frac{s(\psi_k)}{\mathrm{vol}(G)}. \tag{3}$$

Without loss of generality, we rescale the weight $\mathbf{X}$ such that the total sum of edge weights is equal to one, *i.e.*, $\mathrm{vol}(G) = \sum_{u,v} x_{u,v} = 1$. We assume $\mathbf{Z} \in \{0, 1\}^{n \times K}$ is a binary indicator matrix where each entry $z_{i,k}$ records the belongingness of the vertex v_i to the partition P_k, *i.e.*, $z_{i,k} = 1$ if vertex v_i belongs to partition P_k (or $\mu_i \in \psi_k$), and $z_{i,k} = 0$ otherwise. Additionally, we define a degree matrix as $\mathbf{D} = \mathrm{diag}(d_1, \ldots, d_n)$, where d_i is the degree or total weight associated with the vertex v_i. Thus, we are able to rewrite the volume $\mathrm{vol}(\cdot)$ and cohesion $s(\cdot)$ as

$$\begin{aligned} \mathrm{vol}(\psi_k) &= \sum_{i=1}^{n} z_{i,k} d_i z_{i,k} = \mathbf{z}_k^T \mathbf{D} \mathbf{z}_k \\ s(\psi_k) &= \sum_{i=1}^{n} z_{i,k} x_{i,j} z_{j,k} = \mathbf{z}_k^T \mathbf{X} \mathbf{z}_k. \end{aligned} \tag{4}$$

Then, the objective in Equation 3 can be rewritten as

$$\mathcal{T}^{(3)}_{\mathrm{opt}} = \arg\min_{\mathcal{T}^{(3)}} \sum_k \left(2\mathbf{z}_k^T \mathbf{X} \mathbf{z}_k \log_2 \mathbf{z}_k^T \mathbf{D} \mathbf{z}_k - \mathbf{z}_k^T \mathbf{X} \mathbf{z}_k \log_2 \mathbf{z}_k^T \mathbf{X} \mathbf{z}_k\right). \tag{5}$$

If we relax $\mathbf{Z}$ to a real continuous space $\mathbf{Y} \in \mathbb{R}^{n \times K}$, our goal becomes finding the minimum of the following function:

$$\mathcal{J}(G; \mathbf{Y}) = \sum_k \left(2\mathbf{y}_k^T \mathbf{X} \mathbf{y}_k \log_2 \mathbf{y}_k^T \mathbf{D} \mathbf{y}_k - \mathbf{y}_k^T \mathbf{X} \mathbf{y}_k \log_2 \mathbf{y}_k^T \mathbf{X} \mathbf{y}_k\right) \tag{6}$$

Obviously, the solution of Equation 6 is non-unique for minimizing $\mathcal{J}$. To narrow the searching space, we further introduce constraints $\|\mathbf{y}_k\| = 1$ or $\mathbf{y}_k^T \mathbf{y}_k = 1$, which turns the objective into

$$\begin{aligned} \min \quad & \mathcal{J}(G; \mathbf{Y}) \\ \text{s.t.} \quad & \mathbf{y}_k^T \mathbf{y}_k = 1, \forall k \end{aligned} \tag{7}$$

By Lagrangian multipliers α, the Lagrangian function $\mathcal{L}$ and the rationale for solving it, we need to solve the following equations in order to solve the minimization problem Equation 7

$$\nabla_{\mathbf{Y}}\mathcal{J} = \sum_k \alpha_k \nabla_{\mathbf{Y}} f_k(\mathbf{Y}) \tag{8}$$

$$\mathbf{y}_k^T\mathbf{y}_k = 1, \forall k. \tag{9}$$

1.2 Approximate solution and candidate selection

By some calculus, Equation 8 can be reduced to the equality of partial derivatives of two sides for all $\mathbf{y}$'s, leading to

$$\left[\frac{2\mathbf{y}_k^T\mathbf{X}\mathbf{y}_k}{\mathbf{y}_k^T\mathbf{D}\mathbf{y}_k \ln 2}\mathbf{D} + \left(2\log_2 \mathbf{y}_k^T\mathbf{D}\mathbf{y}_k - \log_2 \mathbf{y}_k^T\mathbf{X}\mathbf{y}_k - \frac{\mathbf{y}_k^T\mathbf{X}\mathbf{y}_k}{\mathbf{y}_k^T\mathbf{X}\mathbf{y}_k \ln 2}\right)\mathbf{X}\right]\mathbf{y}_k = \alpha_k\mathbf{y}_k \text{ for } \forall k. \tag{10}$$

Each unit $\mathbf{y}$ for some α that satisfying Equation 10 will be a solution to the minimization problem Equation 7. Instead of solving for $\mathbf{y}$ analytically, we adopted a heuristic method to approximate $\mathbf{y}$.

Note that if we perform some refactoring and substitute with β and λ defined as follows

$$\begin{aligned}\beta &= -\frac{2\left(\mathbf{y}_k^T\mathbf{X}\mathbf{y}_k\right)^2\ln 2}{\mathbf{y}_k^T\mathbf{D}\mathbf{y}_k\left(2\mathbf{y}_k^T\mathbf{X}\mathbf{y}_k \ln 2 \log_2 \mathbf{y}_k^T\mathbf{D}\mathbf{y}_k - \mathbf{y}_k^T\mathbf{X}\mathbf{y}_k \ln 2 \log_2 \mathbf{y}_k^T\mathbf{X}\mathbf{y}_k - \mathbf{y}_k^T\mathbf{X}\mathbf{y}_k\right)} \\ \lambda &= -\frac{\alpha_k\mathbf{y}_k^T\mathbf{X}\mathbf{y}_k \ln 2}{2\mathbf{y}_k^T\mathbf{X}\mathbf{y}_k \ln 2 \log_2 \mathbf{y}_k^T\mathbf{D}\mathbf{y}_k - \mathbf{y}_k^T\mathbf{X}\mathbf{y}_k \ln 2 \log_2 \mathbf{y}_k^T\mathbf{X}\mathbf{y}_k - \mathbf{y}_k^T\mathbf{X}\mathbf{y}_k}\end{aligned} \tag{11}$$

we can rewrite Equation 10 to

$$(\beta\mathbf{D} - \mathbf{X})\,\mathbf{y}_k = \lambda\mathbf{y}_k \tag{12}$$

which implies $\mathbf{y}_k$ is an eigenvector of $\beta\mathbf{D} - \mathbf{X}$. By discretizing the value range of β in lieu of solving for the exact value, we can apply the eigendecomposition on $\mathbf{M}_\beta = \beta\mathbf{D} - \mathbf{X}$ with a given β to obtain eigenvectors $\hat{\mathbf{Y}} = (\hat{\mathbf{y}}_1, \ldots, \hat{\mathbf{y}}_n)$ as the approximate solutions of Equation 10 with corresponding eigenvalues $\lambda_1 \le \lambda_2 \le \cdots \le \lambda_n$.

It can be observed that if $\beta = 1$, Equation 12 will turn into the formulation of the spectral clustering where $\mathbf{D} - \mathbf{X}$ is the Laplacian matrix of $\mathbf{X}$. In this context, the eigenvector $\hat{\mathbf{y}}_2$ corresponding to the first non-zero eigenvalue λ_2, also known as Fiedler vector, divides V into two partitions [1] according to signs of values of $\hat{\mathbf{y}}_2$. Here, we extended the same idea to other eigenvectors. Besides the Fiedler vector, therefore, each eigenvector can be used to assign vertices into a partition set which may be different from those obtained by other eigenvectors (Supplementary Figure 1A-B). Each of the two partitions obtained from each eigenvector may represent or include a true partition, but the quality of the partition degrades as the eigenvalue increases (Supplementary Figure 1C). Furthermore, it appears that smaller true partitions can be obtained by intersecting and complementing partition sets from different eigenvectors (Supplementary Figure 1A). For each β, in fact, we can compute a $\hat{\mathbf{Y}}_\beta$ and hence obtain different partition sets $\hat{\mathbf{Z}}_\beta$. True partitions may be included in these partition sets, as a standalone partition, an intersection, or a complement between partitions. With the whole partition set $\hat{\mathcal{Z}} = \{\hat{\mathbf{Z}}_\beta | \beta \in [\beta_l, \beta_u]\}$ where β_l and β_u are predetermined lower and upper bounds, therefore, we are able to perform intersections and complements between each pair of partition sets to maintain a candidate indicator matrix $\hat{\mathbf{Z}} = (\hat{\mathbf{z}}_1, \ldots, \hat{\mathbf{z}}_N) \in \{0,1\}^{n\times N}$ for N candidate partitions.

$\hat{\mathbf{Z}}$ allows us to identify an optimal set of partitions, $\mathcal{P}_{\text{opt}}$, where each node in V is assigned to one partition and the associated total entropy is minimized. By examining Equation 6, we can observe that for a given $\mathbf{Z}$, each partition contributes independently to the entropy of $\mathcal{T}^{(3)}$. This suggests an additive property and allows the application of integer linear programming (ILP) techniques. Given a candidate matrix $\hat{\mathbf{Z}}$, our goal is to find a set (of column vectors) from candidates such that the following conditions are satisfied: 1) each vertex belongs to exactly one partition; 2) the total entropy of the finally selected partition set is minimized. Let $b_j = \{0,1\}$ be the indicator determining whether $\hat{\mathbf{z}}_j$ is included in the optimal set. Then, the first condition implies that for each vertex the sum of corresponding entries in eventually included candidate vectors should be equal to one, which is interpreted as $\sum_{j=1}^N b_j\hat{z}_{i,j} = 1, \forall i$. For the second condition, we first compute the corresponding entropy contributed by each candidate $\hat{\mathbf{z}}_j$ as $e_j = 2\hat{\mathbf{z}}_j^T\mathbf{X}\hat{\mathbf{z}}_j \log_2 \hat{\mathbf{z}}_j^T\mathbf{D}\hat{\mathbf{z}}_j - \hat{\mathbf{z}}_j^T\mathbf{X}\hat{\mathbf{z}}_j \log_2 \hat{\mathbf{z}}_j^T\mathbf{X}\hat{\mathbf{z}}_j$

and then denote $\mathbf{e} = (e_1, \dots, e_N)^T \in \mathbb{R}^{N\times 1}$. Hence, we can represent the total entropy of selected candidates as $\mathbf{b}^T\mathbf{e}$ with $\mathbf{b} = (b_1, \dots, b_N)^T \in \{0,1\}^{N\times 1}$. As a result, our objective in the ILP form is

$$\begin{aligned} \min \quad & \mathbf{b}^T\mathbf{e} \\ \text{s.t.} \quad & \mathbf{Z}^{\mathcal{C}}\mathbf{b} = \mathbf{1} \end{aligned} \tag{13}$$

where $\mathbf{1}$ is a column vector with all entries as one. Eventually, columns of $\hat{\mathbf{Z}}$ with $b = 1$ compose of $\mathcal{P}_{\text{opt}}$ minimizing the entropy among all candidates given β is in the predetermined range.

1.3 Greedy implementation

Since the number of partition candidates will rapidly increase, as mentioned in the main article, the time complexity will grow accordingly. To address this problem, we applied a greedy method when implementing our CeiTEA.

Recall that for each β, each eigenvector can potentially contribute to a partition set. Now instead of taking all eigenvectors into consideration, we only use n_e eigenvectors for future candidates where n_e is a predetermined parameter of CeiTEA. With the observation in Supplementary Figure 1C that the entropy may decline first and then increase as the eigenvalue increases, we heuristically select the eigenvectors that result in partitions with n_e lowest entropy values and their corresponding partition vectors, which we denote as $\hat{\mathbf{Z}}_\beta = \{\hat{\mathbf{Z}}_{\beta,1}, \dots, \hat{\mathbf{Z}}_{\beta,n_e}\}$ where the entropy values are non-decreasingly sorted and $\hat{\mathbf{Z}}_{\beta,i}$ is the binary indicator matrix corresponding to the partitions by the i-th eigenvector. Then, we greedily estimate the best partitioning by $\hat{\mathbf{Z}}_\beta$ using the following procedures: 1) perform intersections and complements among the candidates provided by $\hat{\mathbf{Z}}_{\beta,1}$ and $\hat{\mathbf{Z}}_{\beta,2}$ to generate the candidate set $\hat{\mathbf{Z}}_{\beta,1\otimes 2}$; 2) use $\hat{\mathbf{Z}}_{\beta,1\otimes 2}$ in ILP to solve for the partitioning with the lowest entropy and obtain the corresponding indicator vectors $\hat{\mathbf{Z}}^*_\beta$; 3) replace $\hat{\mathbf{Z}}_{\beta,1}$ and $\hat{\mathbf{Z}}_{\beta,2}$ with $\hat{\mathbf{Z}}^*_\beta$ and repeat from 1) until the change of entropy values of adjacent iterations is no more than a tolerance (e.g., 10^{-6}). The final $\hat{\mathbf{Z}}^*_\beta$ is the best partitioning for the given β. Then, we can compute such a $\hat{\mathbf{Z}}_\beta$ for each β in a predetermined range bounded by β_l and β_u and also sort them by their entropy values non-increasingly to maintain the candidate set $\hat{\mathbf{Z}}^* = \{\hat{\mathbf{Z}}^*_\beta | \beta \in [\beta_l, \beta_u]\}$. With $\hat{\mathbf{Z}}^*$, finally, we again perform the same procedures producing $\hat{\mathbf{Z}}^*_\beta$ to generate an ultimate partitioning for the given graph.

1.4 Construction of a stratified hierarchy

The optimal partition set $\mathcal{P}_{\text{opt}}$ obtained in the previous section is a plain partitioning, or a three-layer hierarchy. To build a hierarchical structure for the graph G, we can perform a bottom-up strategy on the obtained $\mathcal{P}_{\text{opt}}$. At first, $\mathcal{P}_{\text{opt}}$ is equivalent to $\mathcal{T}^{(3)}_{\text{opt}}$ where the leaves encode graph vertices, internal nodes encode the actual partitions with $b = 1$, and the root encodes the whole graph G. Now, we treat the internal nodes as new leaves and recompute a new weight matrix $\mathbf{X}'$ where each row and column correspond to one internal node of $\mathcal{T}^{(3)}_{\text{opt}}$ (or one partition in $\mathcal{P}_{\text{opt}}$). Thus, we apply the same procedure yielding $\mathcal{P}_{\text{opt}}$ on $\mathbf{X}'$ to partition $\mathbf{X}'$. This process repeats until the partitioning either stabilizes, remaining unchanged from the previous iteration, or results in two partitions. Ultimately, this yields a multi-nary tree, denoted as $\mathcal{T}_{\text{opt-bt}}$.

Following the construction of the optimal $\mathcal{T}_{\text{opt-bt}}$, a top-down approach can be additionally employed to iteratively expand the tree, thereby identifying smaller possible hierarchical structures. This procedure, which we refer to as *local diversification*, involves a systematic exploration of substructures within subtrees, facilitating a more nuanced understanding of underlying hierarchical relationships. For every child node μ, starting from the root node, we extract all leaf nodes encoded by μ, recompute the weight matrix, find the corresponding candidate set, solve the associated ILP for a locally optimal multi-nary tree and replace μ with the new tree. The local diversification along each branch terminates when the associated entropy of the partition from ILP is no smaller than a predetermined value (e.g., zero) to guarantee adequate entropy and hierarchy. In this way, a more stratified hierarchy is produced.

1.5 Estimation of the β range

The quality and number of finally obtained partitions are highly related to the β_l and β_u used for $\hat{\mathcal{Z}}$. By observing the formula for β in Equation 11, we can computationally estimate the numerical range of β under the assumption that $\mathbf{y}_k^T\mathbf{y}_k = 1$. By Rayleigh quotient, the quadratic forms with repecct to the symmetric

$\mathbf{X}$ and $\mathbf{D}$ satisfy the following

$$
\begin{aligned}
x &= \mathbf{y}_k^T \mathbf{X} \mathbf{y}_k = \frac{\mathbf{y}_k^T \mathbf{X} \mathbf{y}_k}{\mathbf{y}_k^T \mathbf{y}_k} \in [\lambda_{\min}(\mathbf{X}), \lambda_{\max}(\mathbf{X})] \\
d &= \mathbf{y}_k^T \mathbf{D} \mathbf{y}_k = \frac{\mathbf{y}_k^T \mathbf{D} \mathbf{y}_k}{\mathbf{y}_k^T \mathbf{y}_k} \in [\lambda_{\min}(\mathbf{D}), \lambda_{\max}(\mathbf{D})]
\end{aligned}
\tag{14}
$$

where $\lambda_{\min}$ and $\lambda_{\max}$ are the minimum and maximum eigenvalues of the corresponding matrix. Since $\mathbf{X}$ and $\mathbf{D}$ are both known with a given graph, we are able to compute the value ranges of x and d and hence we can estimate the range of β by enumerating x and d along their ranges. Depending on the graph, however, the range of β can be potentially too large to be enumerated for computing the whole partition set $\hat{\mathcal{Z}}$ due to the computational complexity. It is necessary to choose a moderate subset from the estimated β range. To have a reference when determining a reasonable range of β, we computed β ranges of all nine single-cell datasets and calculated three metrics based on the eigenvectors with each β value: (1) the minimum topological entropy among the partitions obtained by all eigenvectors (like the Fiedler vector); (2) the maximum adjusted Rand Index (ARI) score among the partitions obtained by all eigenvectors; (3) the maximum correlation coefficient between the truth dummy labels and all eigenvectors. For (1) and (2), the partitions were obtained by the similar process that the spectral clustering used to divide nodes into two groups by the Fiedler vector. For (3), the correlation coefficients were calculated as follows. At first, we converted the truth labels to a matrix form which was exactly the same as $\mathbf{Z}$. Then, for each dummy label we calculated the correlation coefficients between the dummy label and all eigenvectors, and computed the maximum one. Finally, for each β we computed the maximum correlation coefficient among all dummy labels.

Supplementary Figure 3 shows changes of the metrics as β shifts in the single-cell datasets. From the peak points for the three metrics, we may conclude that by choosing β around zero (e.g. 0-10) we could obtain more consistent or reliable partitions from eigenvectors since the partitions from that range likely have lower topological entropy, higher ARI scores and correlations and are more probable to include the true partitions or generate reliable partition intersection and hence a more meaningful partition set. For most samples, the range $(0, 1]$ was enough to include the possible good partition candidates as the minimum topological entropy values were observed with $\beta \leq 1$. For a more robust result as well as the consideration that the ground truths are usually absent in real world, therefore, we chose $(0, 2]$ as the parameter for β in this study.

2 Supplementary Tables

group	Term	Genes	Adjusted P-value
D0-Pluripotent cells (2)	Endodermal Cell Fate Commitment	SOX2;POU5F1	1.77e-02
D0-Pluripotent cells (3)	Pituitary Gland Development	SOX2;HESX1	4.59e-02
D1-Anterior PS (0)	Gastrulation	EOMES;CER1;MIXL1	3.00e-03
D1-Anterior PS (0)	Epidermal Cell Differentiation	GATA6;PITX2;EPHA2	1.95e-02
D1-Anterior PS (0)	Cardiocyte Differentiation	BMP2;GATA6	2.00e-02
D1-Anterior PS (1)	Gastrulation	EOMES;CER1;MIXL1	5.91e-03
D1-Anterior PS (2)	Gastrulation	EOMES;CER1;MIXL1	3.49e-03
D1-Anterior PS (2)	Cardiocyte Differentiation	BMP2;GATA6	2.20e-02
D1-Anterior PS (2)	Endoderm Development	EOMES;MIXL1	2.20e-02
D1-Anterior PS (3)	Gastrulation	EOMES;CER1;MIXL1	4.23e-03
D1-Anterior PS (3)	Inhibitory Synapse Assembly	GABRB2;FGF13	2.49e-02
D1-Anterior PS (3)	Endoderm Development	EOMES;MIXL1	2.52e-02
D1-Anterior PS (4)	Gastrulation	EOMES;CER1;MIXL1	7.06e-03
D1-Anterior PS (4)	Cardiocyte Differentiation	BMP2;GATA6	3.98e-02
D1-Anterior PS (4)	Endoderm Development	EOMES;MIXL1	3.98e-02
D2-Lateral mesoderm (0)	Chordate Embryonic Development	FOXF1;WNT5A;NOG;GATA3;ISL1	9.80e-05
D2-Lateral mesoderm (0)	Ventricular Cardiac Muscle Tissue Development	IRX3;ID2;HAND1;ISL1	9.80e-05
D2-Lateral mesoderm (0)	Regulation Of Osteoblast Differentiation	PPP3CA;BAMBI;NOG;PRKD1;SMAD6	1.74e-04
D2-Lateral mesoderm (1)	Chordate Embryonic Development	FOXF1;WNT5A;NOG;GATA3;ISL1	2.30e-04
D2-Lateral mesoderm (1)	Regulation Of Osteoblast Differentiation	PPP3CA;BAMBI;HGF;NOG;PRKD1	5.76e-04
D2-Lateral mesoderm (1)	Pharyngeal System Development	NOG;GATA3;ISL1	8.57e-04
D2-Lateral mesoderm (2)	Regulation Of Osteoblast Differentiation	PPP3CA;BAMBI;NOG;PRKD1;SMAD6	4.62e-04
D2-Lateral mesoderm (2)	Pharyngeal System Development	NOG;GATA3;ISL1	7.37e-04
D2-Lateral mesoderm (2)	Chordate Embryonic Development	FOXF1;NOG;GATA3;ISL1	9.11e-04
D2-Lateral mesoderm (3)	Regulation Of Osteoblast Differentiation	PPP3CA;BAMBI;NOG;PRKD1;SMAD6	3.73e-04
D2-Lateral mesoderm (3)	Chordate Embryonic Development	FOXF1;WNT5A;NOG;ISL1	1.35e-03
D2-Lateral mesoderm (3)	Endocardial Cushion Development	FOXF1;NOG;ISL1	2.09e-03
D2-Paraxial mesoderm (0)	Regulation Of Nervous System Development	HES6;GBX2;LEF1	1.89e-02
D2-Paraxial mesoderm (0)	Positive Regulation Of Glial Cell Differentiation	EGR2;CXCR4	4.05e-02
D2-Paraxial mesoderm (1)	Positive Regulation Of Glial Cell Differentiation	EGR2;CXCR4	4.49e-02
D2-Paraxial mesoderm (1)	Mesoderm Development	HES7;TBX6	4.69e-02
D2-Paraxial mesoderm (2)	Regulation Of Nervous System Development	HES7;HES6;GBX2;LEF1	6.78e-04
D2-Paraxial mesoderm (2)	Mesoderm Development	HES7;TBX6;MEST	1.20e-03
D2-Paraxial mesoderm (2)	Nose Development	PROX1;STRA6	6.78e-03
D2-Paraxial mesoderm (3)	Regulation Of Nervous System Development	HES7;HES6;LEF1	2.21e-02
D2-Paraxial mesoderm (3)	Regulation Of Cell Development	HES7;HES6;LEF1	2.21e-02
D2-Paraxial mesoderm (3)	Endothelial Cell Development	F2RL1;ICAM1	4.07e-02
D2.25-Somitomeres (0)	Regulation Of Neuron Differentiation	HEYL;PCP4;DLL1;RHOA	1.02e-02
D2.25-Somitomeres (0)	Skeletal Muscle Thin Filament Assembly	ACTC1;TTN	1.02e-02
D2.25-Somitomeres (0)	Substantia Nigra Development	DYNLL1;CALM1;RHOA	1.02e-02
D2.25-Somitomeres (1)	Skeletal Muscle Thin Filament Assembly	ACTC1;TTN	5.99e-03
D2.25-Somitomeres (1)	Skeletal Myofibril Assembly	ACTC1;TTN	7.96e-03
D2.25-Somitomeres (1)	Regulation Of Cell Development	HEYL;ANXA2;DLL1	1.09e-02
D2.25-Somitomeres (2)	Regulation Of Cell Development	HEYL;ANXA2;DLL1;S100A10	1.63e-03
D2.25-Somitomeres (2)	Regulation Of Neuron Differentiation	HEYL;PCP4;DLL1;RHOA	4.63e-03
D2.25-Somitomeres (2)	Positive Regulation Of Neuron Differentiation	HEYL;PCP4;RHOA	9.32e-03
D3-Early somites (0)	Mesonephric Tubule Development	SIX1;FGFR2	3.61e-02
D3-Early somites (0)	Mesenchyme Development	MEOX1;FGFR2	3.61e-02
D3-Early somites (0)	Ureteric Bud Development	SIX1;FGFR2	3.62e-02
D3-Early somites (1)	Skin Development	COL1A1;ANXA1;COL1A2;PDGFA	7.52e-03
D3-Early somites (1)	Skeletal Muscle Organ Development	SVIL;MYL6;SIX1	9.28e-03
D3-Early somites (1)	Muscle Tissue Development	SVIL;MYL6;SIX1	9.28e-03
D5-Dermomyotome (1)	Positive Regulation Of Dendrite Development	PTN;BMP5	2.71e-02
D5-Dermomyotome (1)	Positive Regulation Of Stem Cell Differentiation	PTN;SOX5	4.41e-02
D5-Dermomyotome (3)	Pharyngeal System Development	NOG;HES1;BMP5	3.86e-03
D5-Dermomyotome (3)	Negative Regulation Of Glial Cell Differentiation	NOG;HES1	2.04e-02
D5-Dermomyotome (3)	Regulation Of Astrocyte Differentiation	NOG;HES1	3.19e-02
D5-Sclerotome (0)	Positive Regulation Of Kidney Development	FOXD1;SOX9	3.02e-03
D5-Sclerotome (0)	Noradrenergic Neuron Differentiation	SOX11;SOX4	3.02e-03
D5-Sclerotome (0)	Ventricular Septum Development	TGFB2;SOX11;SOX4	3.34e-03
D5-Sclerotome (1)	Noradrenergic Neuron Differentiation	SOX11;SOX4	3.54e-03
D5-Sclerotome (1)	Ventricular Septum Development	TGFB2;SOX11;SOX4	3.70e-03
D5-Sclerotome (1)	Neuroepithelial Cell Differentiation	SOX11;SOX4	4.19e-03
D5-Sclerotome (2)	Ventricular Septum Development	TGFB2;HES1;SOX11;SOX4	3.03e-04
D5-Sclerotome (2)	Regulation Of Osteoblast Differentiation	FBN2;PTCH1;TWIST1;SOX11;SOX9	3.03e-04
D5-Sclerotome (2)	Pharyngeal System Development	TGFB2;PTCH1;HES1	8.16e-04
D5-Sclerotome (3)	Regulation Of Osteoblast Differentiation	FBN2;PTCH1;TWIST1;SOX11;SOX9;BMPR1B	2.91e-05
D5-Sclerotome (3)	Positive Regulation Of Stem Cell Differentiation	TGFB2;PTN;SOX9	8.44e-04
D5-Sclerotome (3)	Negative Regulation Of Osteoblast Differentiation	PTCH1;TWIST1;SOX9	3.15e-03

Table 1: Top three GO terms results from Layer 5 clusters in the Koh dataset.

group	Term	Genes	Adjusted P-value
3_8cell_1	Cytoplasmic Translation	EIF4A1;RPS7;RPL27A;RPL24;RPL38;RPL26;RPL29;RPS21	2.43e-08
	Positive Regulation Of Intrinsic Apoptotic Signaling Pathway By P53 Class Mediator	RPS7;RPL26	3.35e-03
	Mitochondrial Electron Transport, Cytochrome C To Oxygen	COX4I1;COX7C	3.39e-02
	Intracellular Lipid Transport	TMEM41B;NPC2	3.77e-02

Table 2: GO results from Layer 2 clusters in the Goolam dataset.

group	Term	Genes	Adjusted P-value
Layer 1: 1	Sema4D Induced Cell Migration And Growth-Cone Collapse R-HSA-416572	ERBB2;MYH9	2.63e-02
Layer 1: 1	Post-chaperonin Tubulin Folding Pathway R-HSA-389977	TUBA1B;TUBB	2.82e-02
Layer 1: 1	RHOBTB2 GTPase Cycle R-HSA-9013418	HSP90AB1;ACTG1	2.82e-02
Layer 1: 1	Sema4D In Semaphorin Signaling R-HSA-400685	ERBB2;MYH9	2.82e-02
Layer 1: 1	Attenuation Phase R-HSA-3371568	HSP90AB1;SERPINH1	2.82e-02
Layer 1: 2	MyD88 Deficiency	S100A1;S100A9	4.06e-02
Layer 1: 2	IRAK4 Deficiency	S100A1;S100A9	4.06e-02
Layer 1: 2	Regulation Of TLR By Endogenous Ligand R-HSA-5686938	S100A1;S100A9	4.06e-02
Layer 1: 2	RHO GTPases Activate CIT R-HSA-5625900	RNASE1;MYL9	4.06e-02
Layer 1: 2	RHO GTPases Activate PAKs R-HSA-5627123	RNASE1;MYL9	4.06e-02
Layer 1: 3	Endosomal/Vacuolar Pathway R-HSA-1236977	HLA-B;HLA-C;HLA-F;B2M	5.74e-06
Layer 1: 3	ER-Phagosome Pathway R-HSA-1236974	PSMB7;HLA-B;HLA-C;HLA-F;B2M;UBA52	1.13e-05
Layer 1: 3	Antigen processing-Cross Presentation R-HSA-1236975	PSMB7;HLA-B;HLA-C;HLA-F;B2M;UBA52	1.92e-05
Layer 1: 3	Antigen Presentation: Folding, Assembly, Peptide Loading Of Class I MHC R-HSA-983170	HLA-B;HLA-C;HLA-F;B2M	3.84e-05
Layer 1: 3	Interferon Alpha/Beta Signaling R-HSA-909733	IFITM3;IFI27;HLA-B;HLA-C;HLA-F	4.87e-05
Layer 2: 1_0	TGF-beta Receptor Signaling Activates SMADs R-HSA-2173789	STUB1;UBA52;PPP1CA;UBE2M	1.08e-03
Layer 2: 1_0	Defective B3GALT6 Causes EDSP2 And SEMDJL1 R-HSA-4420332	BGN;SDC1;HSPG2	1.48e-03
Layer 2: 1_0	Defective B3GAT3 Causes JDSSDHD R-HSA-3560801	BGN;SDC1;HSPG2	1.48e-03
Layer 2: 1_0	Defective B4GALT7 Causes EDS, Progeroid Type R-HSA-3560783	BGN;SDC1;HSPG2	1.48e-03
Layer 2: 1_0	Plasma Lipoprotein Assembly, Remodeling, And Clearance R-HSA-174824	AP2S1;P4HB;APOE;UBA52	2.23e-03
Layer 2: 1_2	Antigen Presentation: Folding, Assembly, Peptide Loading Of Class I MHC R-HSA-983170	HLA-B;HLA-A;B2M;TAPBP	6.80e-05
Layer 2: 1_2	Endosomal/Vacuolar Pathway R-HSA-1236977	HLA-B;HLA-A;B2M	1.77e-04
Layer 2: 1_2	ER-Phagosome Pathway R-HSA-1236974	UBC;HLA-B;HLA-A;B2M;TAPBP	1.77e-04
Layer 2: 1_2	Interferon Gamma Signaling R-HSA-877300	HLA-B;HLA-DRA;HLA-A;B2M;HLA-DRB1	1.77e-04
Layer 2: 1_2	Modulation By Mtb Of Host Immune System R-HSA-9637628	UBC;B2M	4.42e-03
Layer 2: 2_0	Endosomal/Vacuolar Pathway R-HSA-1236977	HLA-B;B2M	1.77e-02
Layer 2: 2_0	Initial Triggering Of Complement R-HSA-166663	C3;C1QA	4.07e-02
Layer 2: 2_0	Antigen Presentation: Folding, Assembly, Peptide Loading Of Class I MHC R-HSA-983170	HLA-B;B2M	4.24e-02
Layer 2: 2_2	Transcriptional Regulation Of White Adipocyte Differentiation R-HSA-381340	FABP4;LEP;ADIPOQ;LPL;ADIRF;CD36;PLIN1	5.04e-07
Layer 2: 2_2	Triglyceride Catabolism R-HSA-163560	LIPE;FABP4;CAV1;PLIN1	4.83e-05
Layer 2: 2_2	Triglyceride Metabolism R-HSA-8979227	LIPE;FABP4;CAV1;PLIN1	2.34e-04
Layer 2: 2_2	Binding And Uptake Of Ligands By Scavenger Receptors R-HSA-2173782	SPARC;COL4A1;CD36;FTL	2.67e-04
Layer 2: 2_2	Classical Antibody-Mediated Complement Activation R-HSA-173623	C1QB;C1QA	3.88e-03
Layer 2: 2_3	Advanced Glycosylation Endproduct Receptor Signaling R-HSA-879415	APP;LGALS3;SAA2	5.97e-04
Layer 2: 2_3	RUNX1 Regulates Transcription Of Genes Involved In Differentiation Of Myeloid Cells R-HSA-8939246	LGALS3;RUNX1	8.18e-03
Layer 2: 2_3	Binding And Uptake Of Ligands By Scavenger Receptors R-HSA-2173782	FTH1;SAA2;JCHAIN	8.18e-03
Layer 2: 2_3	Formyl Peptide Receptors Bind Formyl Peptides And Many Other Ligands R-HSA-444473	APP;SAA2	8.18e-03
Layer 2: 2_3	Ephrin Signaling R-HSA-3928664	RNASE1;EPHB3	2.95e-02
Layer 2: 3_0	Smooth Muscle Contraction R-HSA-445355	MYL6;ANXA6;MYL9	1.13e-02
Layer 2: 3_0	Endosomal/Vacuolar Pathway R-HSA-1236977	HLA-B;HLA-C	1.63e-02
Layer 2: 3_0	EPHA-mediated Growth Cone Collapse R-HSA-3928663	MYL6;MYL9	2.67e-02
Layer 2: 3_0	RHO GTPases Activate ROCKs R-HSA-5627117	MYL6;MYL9	3.17e-02
Layer 2: 3_0	RHO GTPases Activate CIT R-HSA-5625900	MYL6;MYL9	3.17e-02
Layer 2: 3_1	Endosomal/Vacuolar Pathway R-HSA-1236977	HLA-C;B2M	1.62e-02
Layer 2: 3_1	Chemokine Receptors Bind Chemokines R-HSA-380108	CCL21;CCL5;CCL19	1.62e-02
Layer 2: 3_1	Scavenging By Class A Receptors R-HSA-3000480	COL1A1;FTL	2.98e-02
Layer 2: 3_1	Antigen Presentation: Folding, Assembly, Peptide Loading Of Class I MHC R-HSA-983170	HLA-C;B2M	4.22e-02
Layer 2: 3_1	EPHB-mediated Forward Signaling R-HSA-3928662	ARPC3;ACTG1	4.86e-02

Table 3: Top five GO terms from clusters in the breast cancer H1 sample.

3 Supplementary Figures

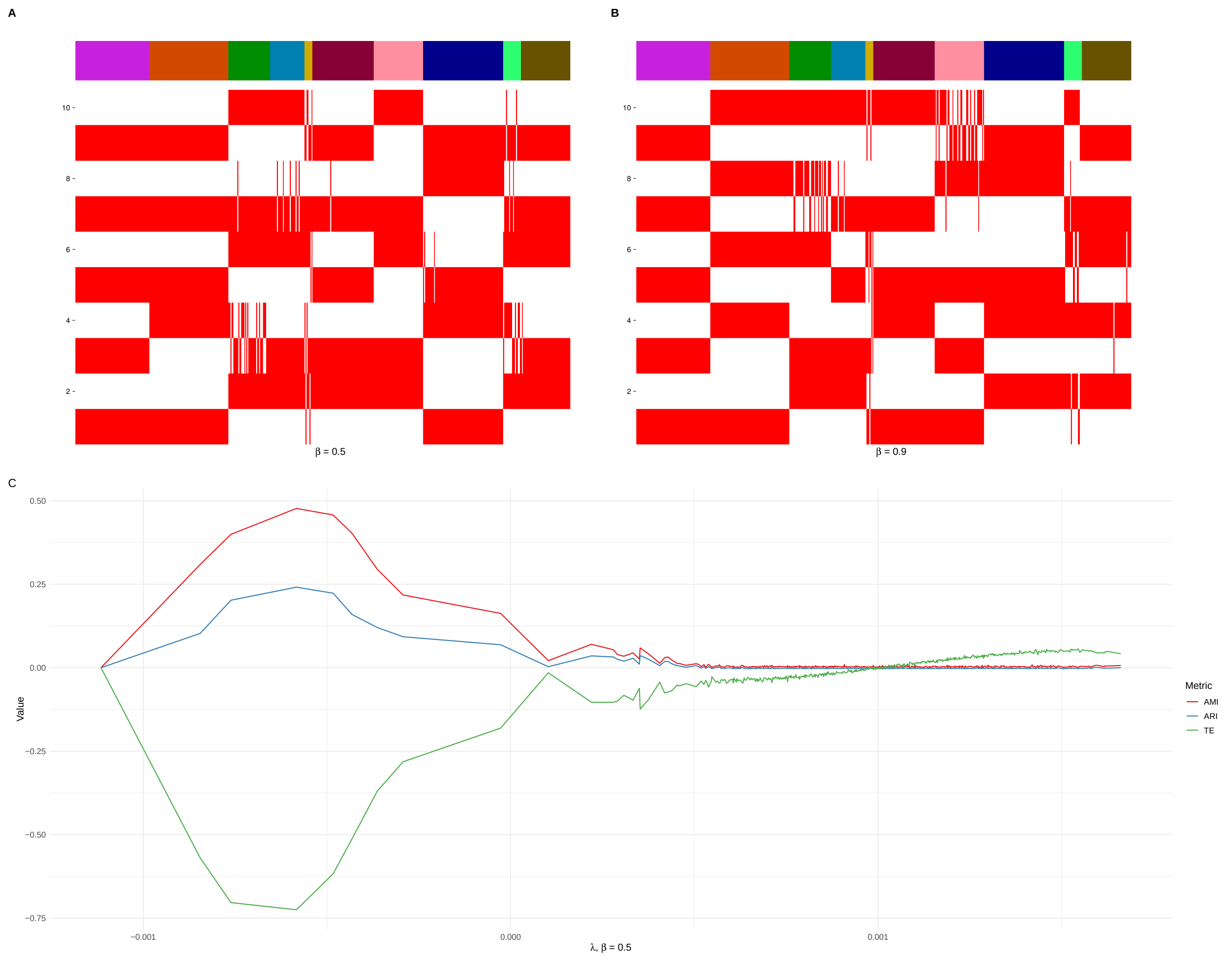

Supplementary Figure 1: Example CeiTEA candidate generation. **A-B.** The X-axis orders leaf nodes and the top color strips are the ground truth clusters. Red colors in the heatmap indicate the corresponding nodes belong to the same partition. From bottom, the labels correspond to the partition candidates from eigenvectors with increasing topological entropy. **A.** Labels from eigenvectors with $\beta = 0.5$. The first and fo will produce the first cluster (purple) by intersection and complement. **B.** Labels from eigenvectors with $\beta = 0.9$. **C.** The change of topological entropy, adjusted Rand Index, and adjusted mutual information as the increase of eigenvalues of $\beta\mathbf{D} - \mathbf{X}$ with $\beta = 0.5$.

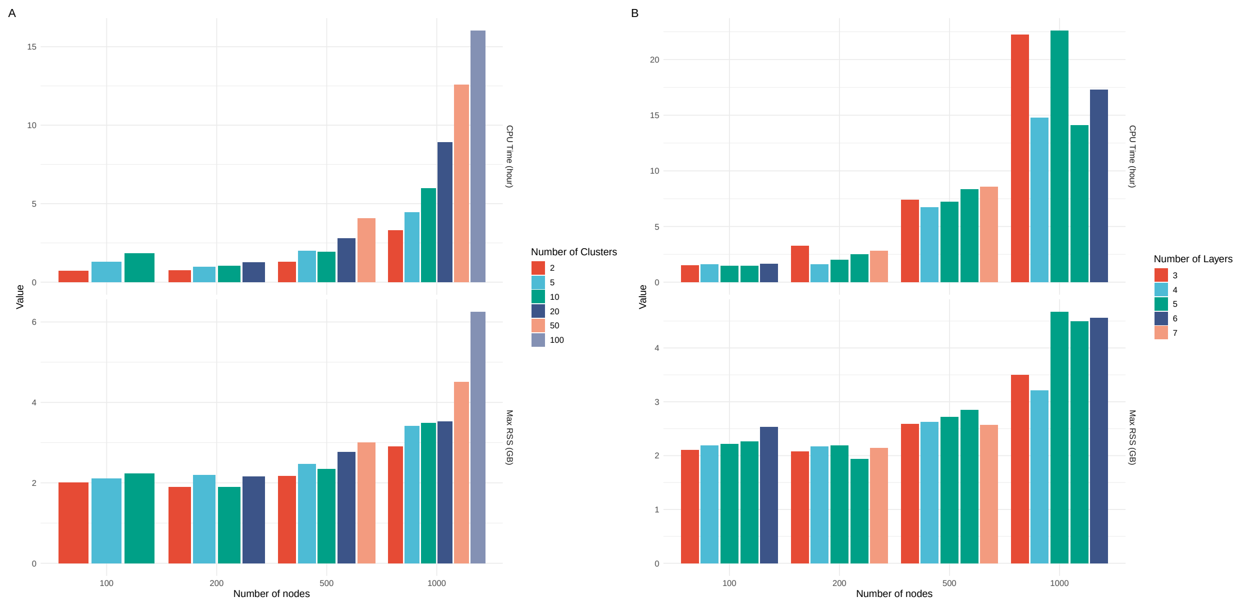

Supplementary Figure 2: Resources used by CeiTEA.

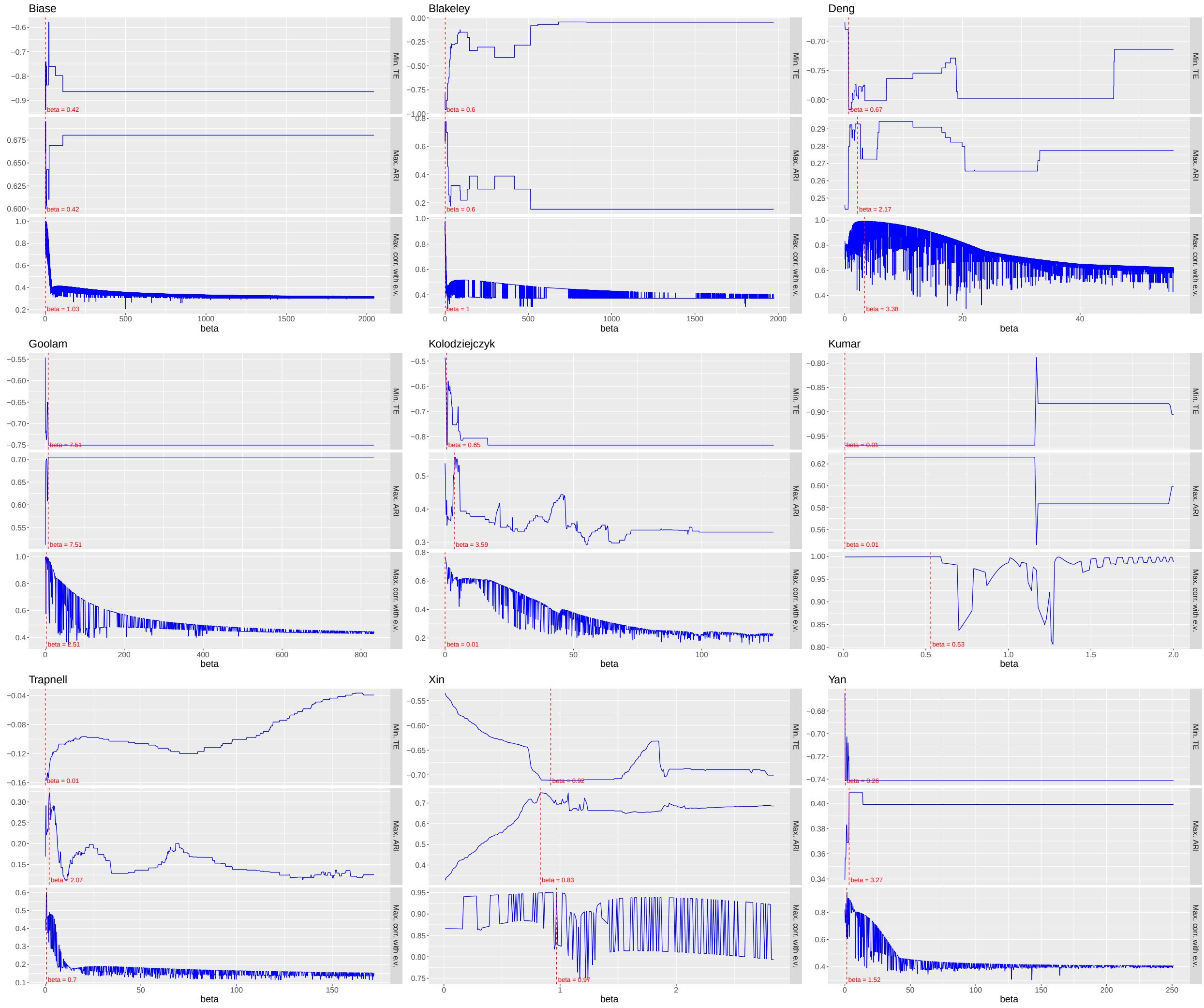

Supplementary Figure 3: The minimum topological entropy of partitions by eigenvectors, maximum adjusted Rand Index of partitions by eigenvectors and maximum correlation coefficient between the truth dummy labels and eigenvectors with each β value in the estimated range. The red dashed vertical line indicates the corresponding β where the minimum or maximum value is obtained.

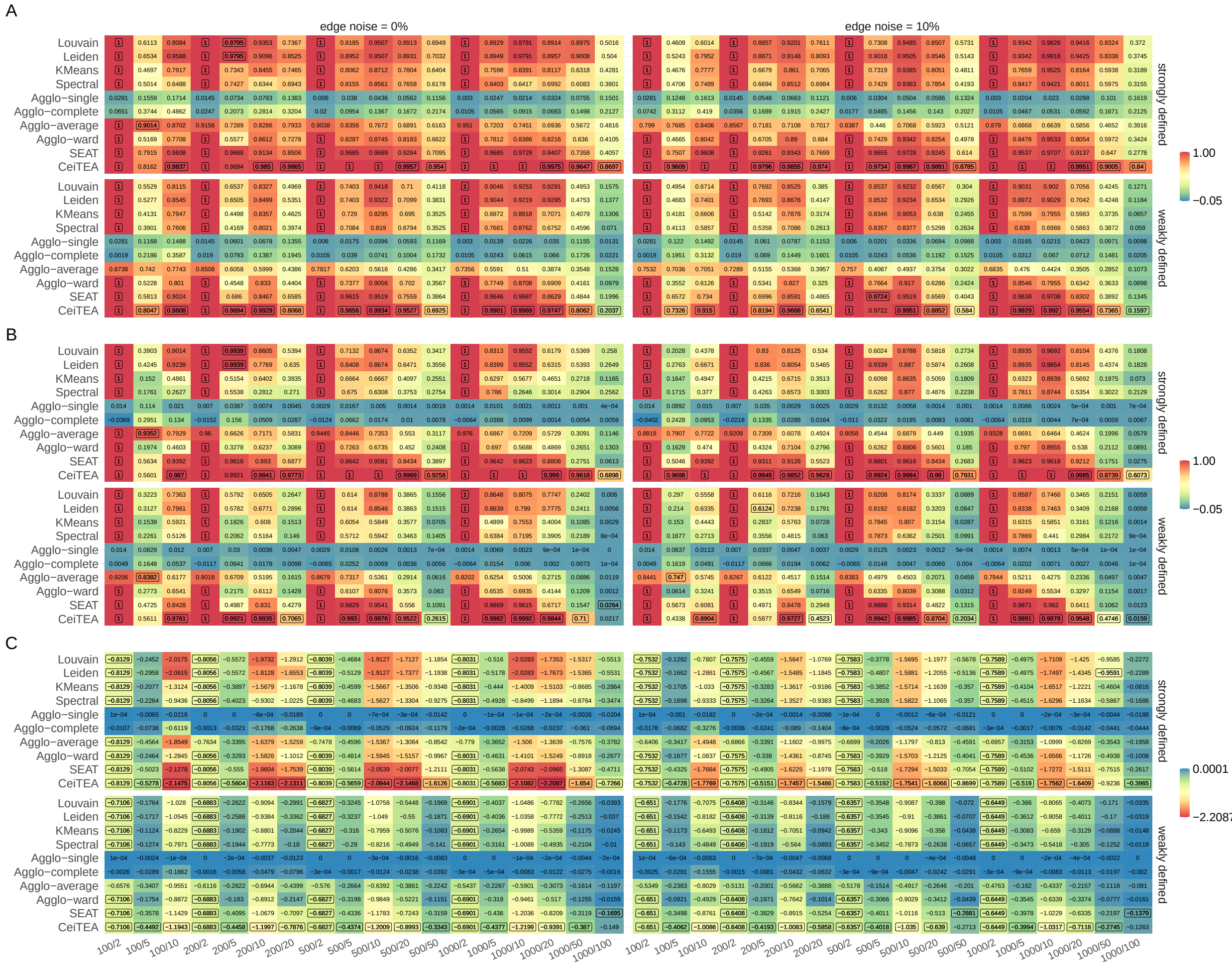

Supplementary Figure 4: Heatmap of ARI, AMI and topological entropy of all methods in simualted datasets across different levels of edge noise and wellness of cluster definitions. **A.** AMI scores across noise and cluster definitions. **B.** ARI scores across noise and cluster definitions. **C.** Topological entropy across noise and cluster definitions. Values surrounded by boxes indicate the maximum (for ARI and AMI) and minimum (for entropy) among all methods.

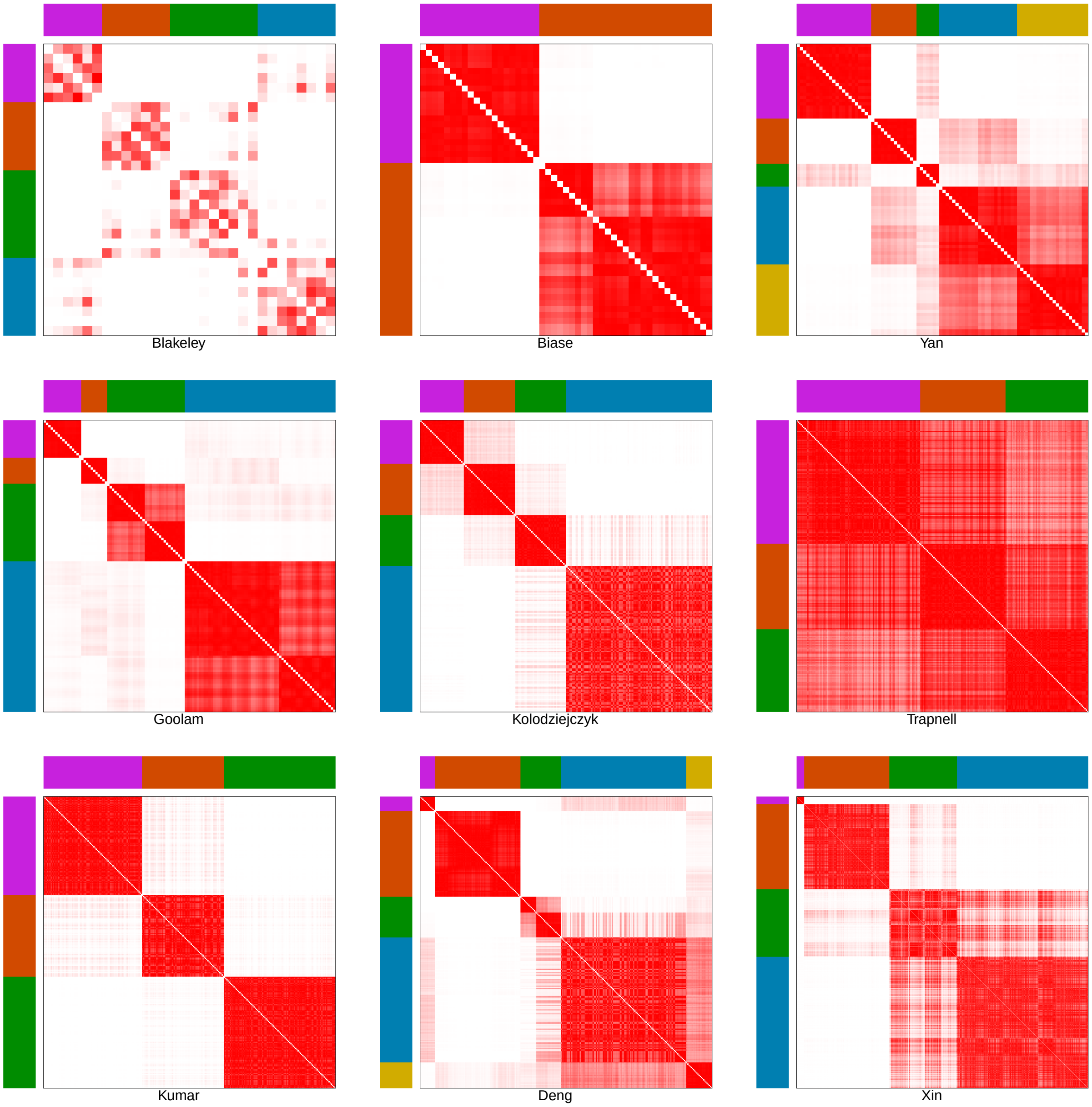

Supplementary Figure 5: Clustering results and the sorted affinity matrices of CeiTEA on real single-cell datasets.

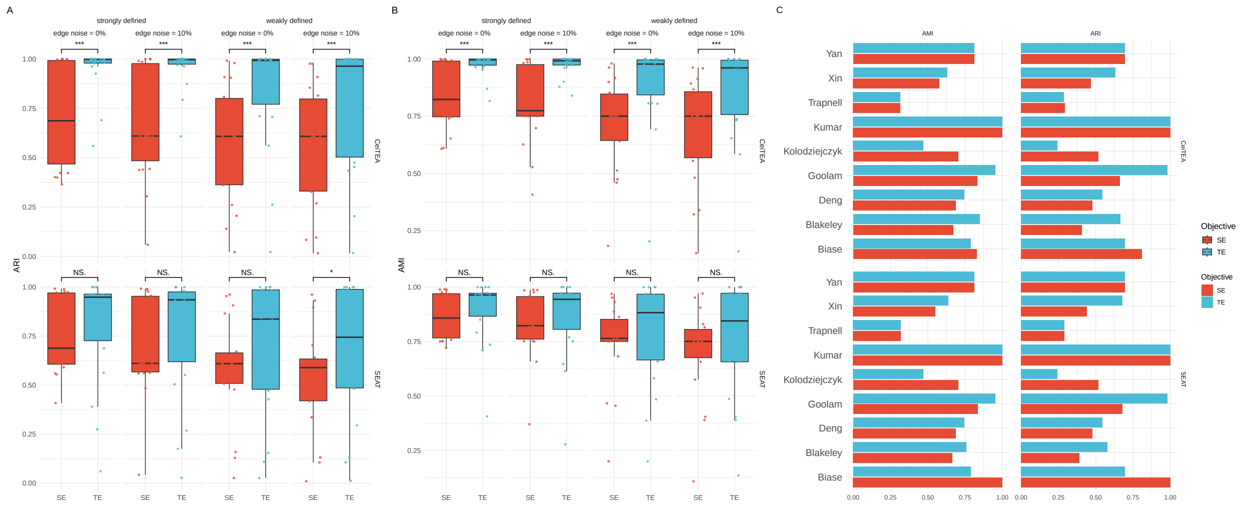

Supplementary Figure 6: Comparisons between SE and TE of CeiTEA and SEAT on single-layer simulation and real datasets.
A. Boxplots of ARI scores of SE and TE objectives from both methods on simulated datasets. **B.** Boxplots of AMI scores of SE and TE objectives from both methods on simulated datasets. **C.** Barplots of ARI and AMI scores of SE and TE objectives from both methods on real single-cell datasets. The significance is calculated using a one-sided paired Wilcoxon test, with the following annotations: **** for p-value ≤ 0.0001, *** for p-value ≤ 0.001, ** for p-value ≤ 0.01, * for p-value ≤ 0.05, and NS for p-value > 0.05.

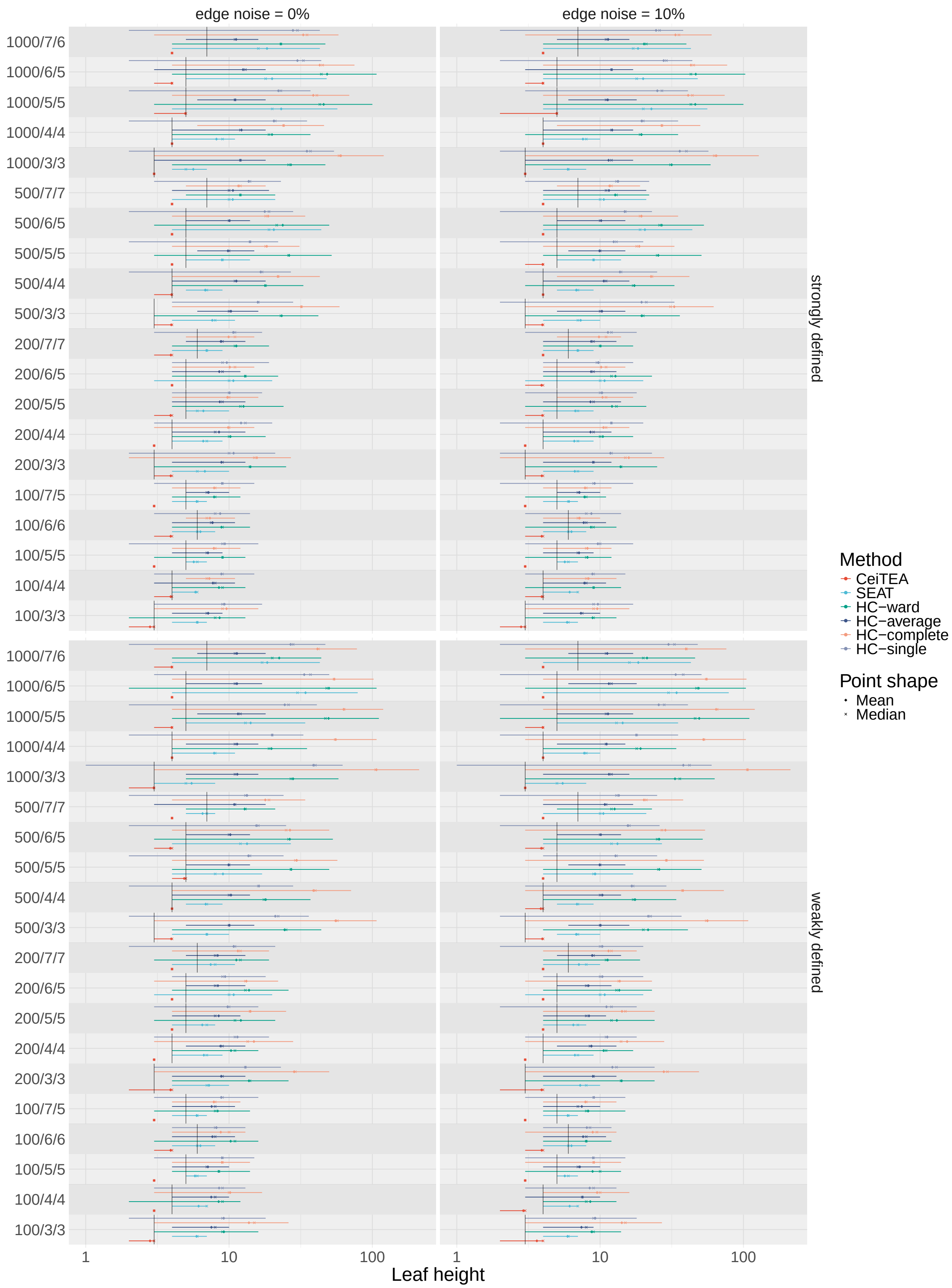

Supplementary Figure 7: An overview of heights of leaf nodes in hierarchies estimated by CeiTEA and other tools. The height of the true hierarchy is depicted as a black vertical line in each row. Row labels in the left are "node size/expected height when simulation/true height after simulation".

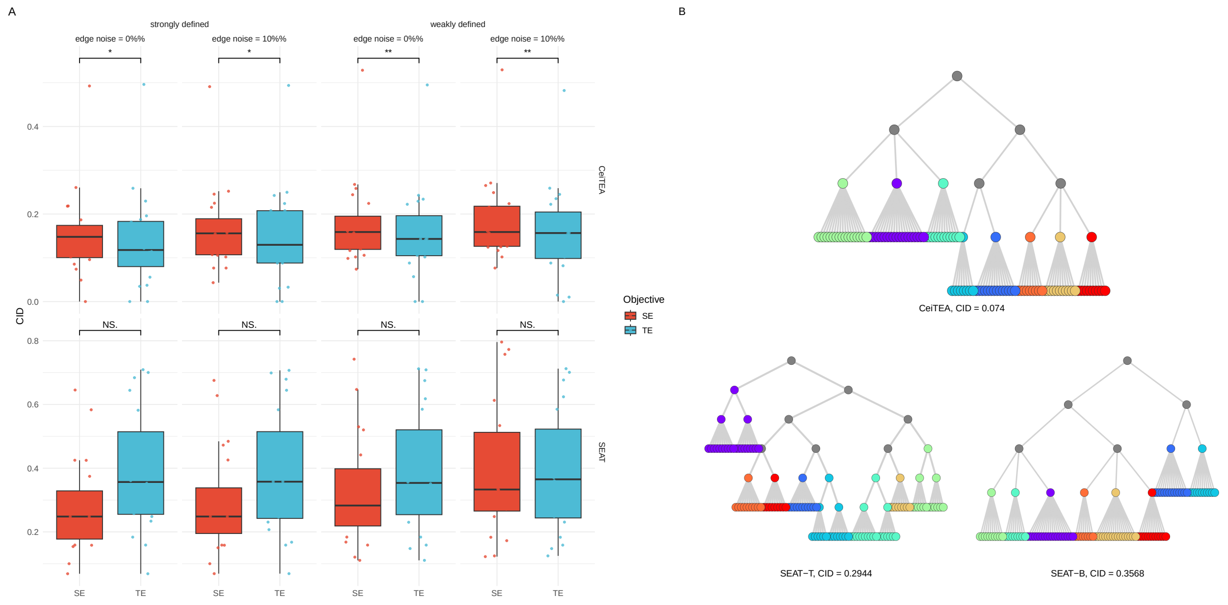

Supplementary Figure 8: Comparisons between SE and TE of CeiTEA and SEAT on simulated multi-layer datasets, and the hierarchical trees based on SE.

A. Boxplots of CID scores of SE and TE objectives from both methods on simulated datasets. **B.** Hierarchical trees of an example simulated dataset, from CeiTEA, SEAT with the top-down strategy (SEAT-T) and SEAT with the bottom-up strategy (SEAT-B). The node color, except for grey, indicates that the children belong to the same partition.

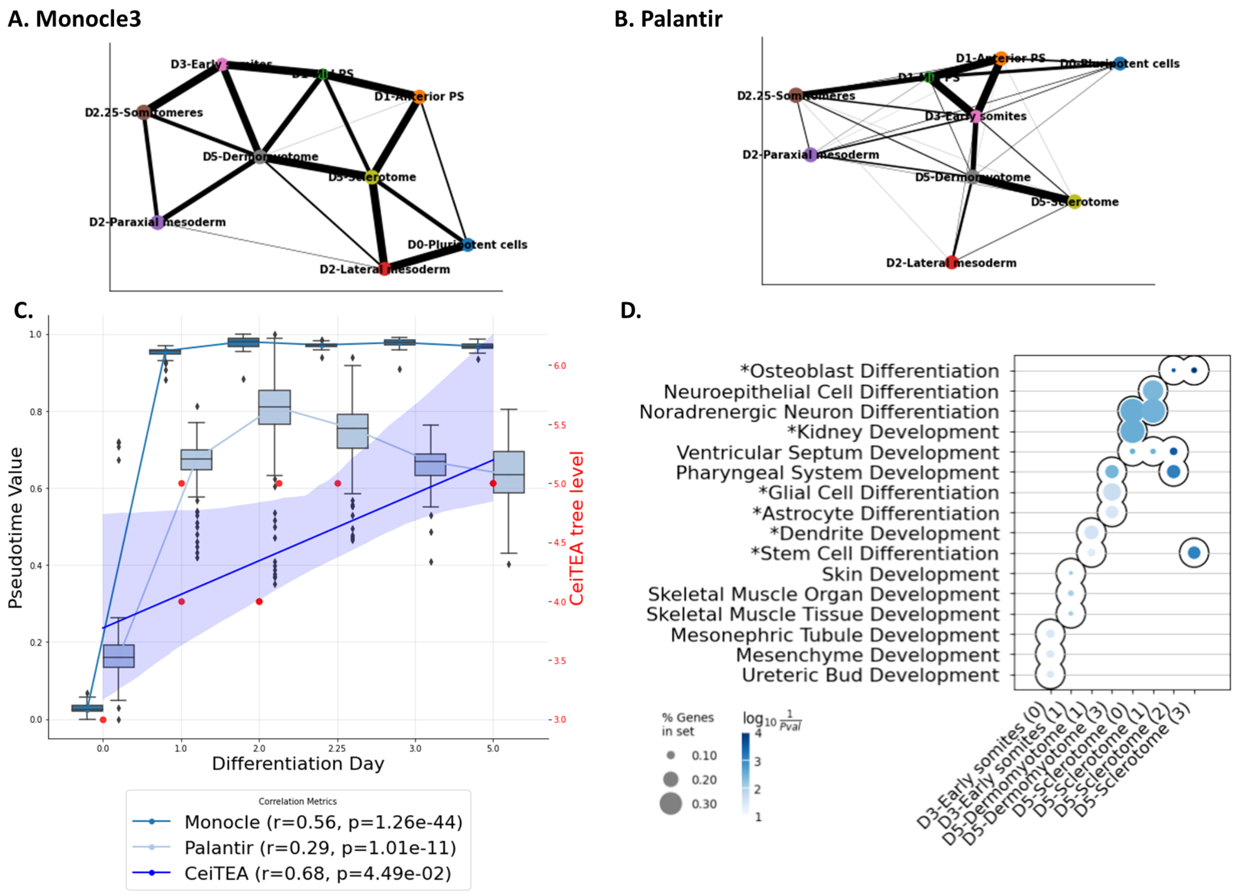

Supplementary Figure 9: Additional results for mesoderm dataset.
A-B. Cell type trajectory from Monocle3 and Palantir. **C.** Boxplots of pseudo-time distribution against differentiation day from Monocle3 and Palantir, overlayed with scatters and a regression line showing the correlation between the differentiation day and the corresponding tree levels from CeiTEA. The legend shows the correlation coefficients and p-values. **D.** The dot plot lists the top three GO terms enriched by the top fifty marker genes in subclusters of Early somites, Dermomyotome, and Sclerotome cells in layer 6.

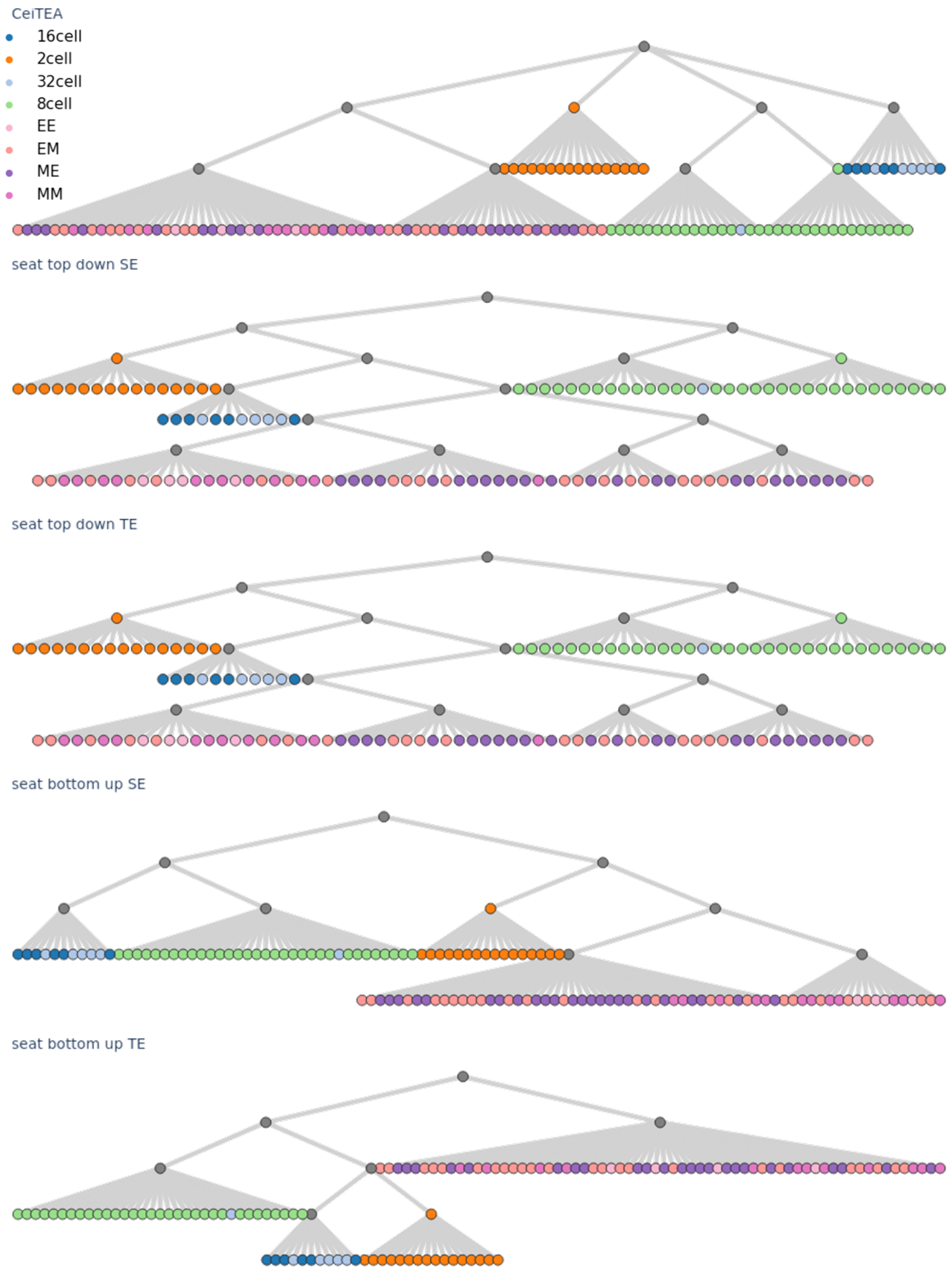

Supplementary Figure 10: Hierarchies of CeiTEA and SEAT.
From top to bottom: CeiTEA, SEAT top-down strategy with SE and TE objective, and SEAT bottom-up strategy with SE and TE objective.

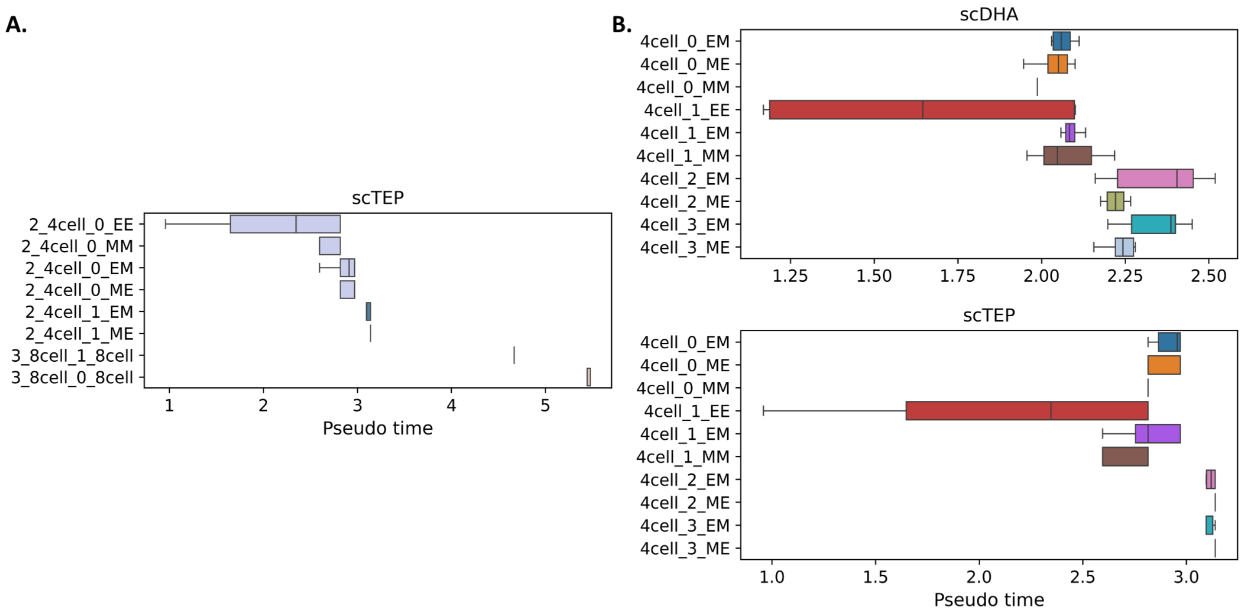

Supplementary Figure 11: Pseudo-time analysis on CeiTEA and SEAT results.
Pseudo-time analysis on results from CeiTEA Layer 2 clusters and SEAT top-down strategy with the TE objective. **A.** Boxplot of scTEP pseudo-time distributions in 4-cell subsets of SEAT. **B.** Boxplot of scTEP and scDHA pseudo-time distributions in 4-cell subsets of SEAT.

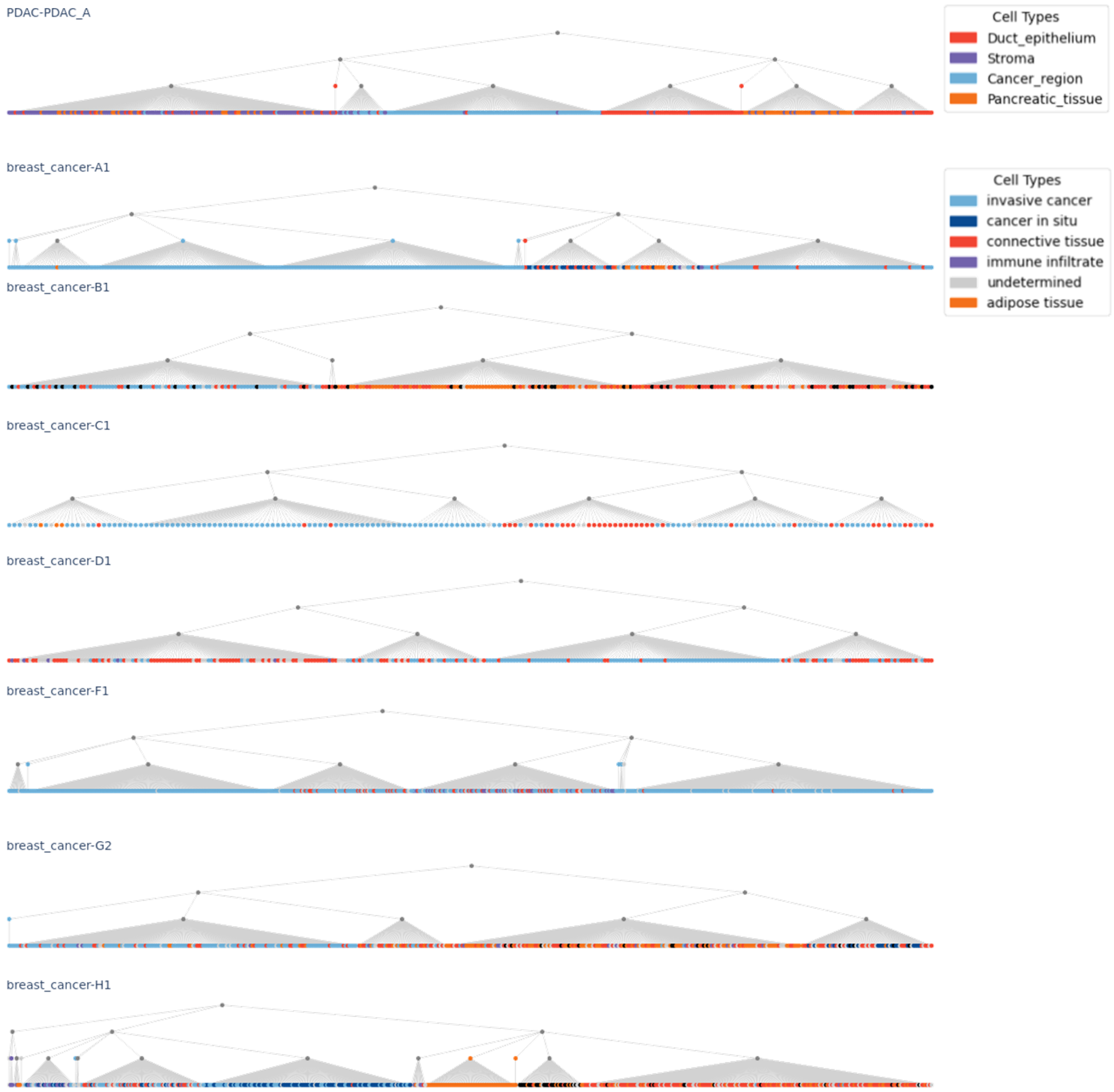

Supplementary Figure 12: Overview of CeiTEA hierarchies on spatial transcriptome samples. Each row showcases the CeiTEA hierarchy of a spatial transcriptome sample.

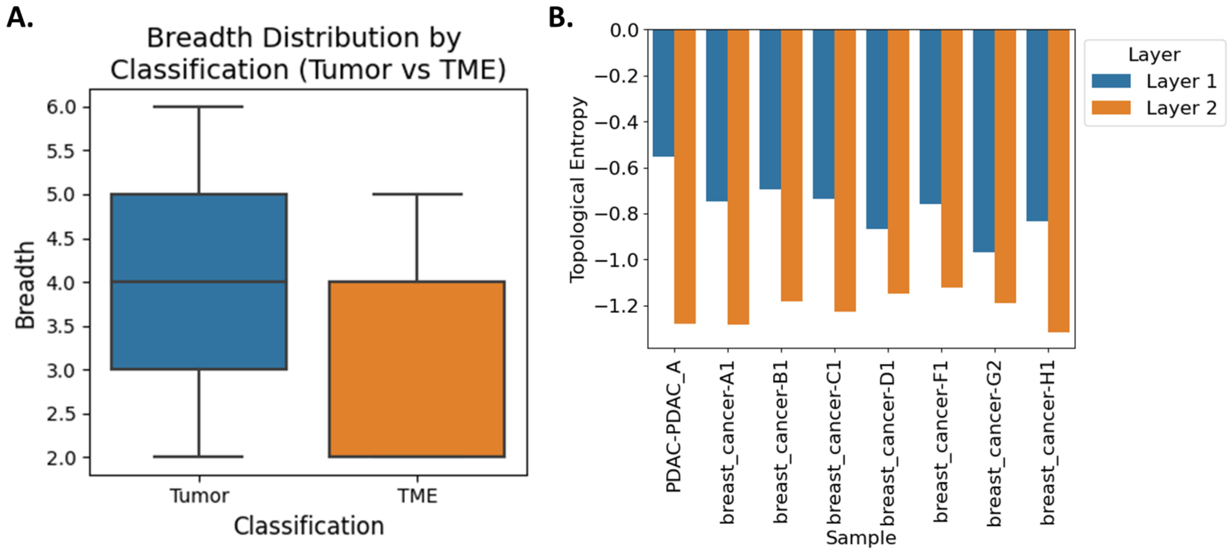

Supplementary Figure 13: Breadth distribution and topological entropy values of CeiTEA hierarchies on spatial transcriptome samples.

A. Breadth distribution in Layer 1 tumor and TME clusters. **B.** Topological entropy values for both layers in CeiTEA hierarchies across samples on both datasets.

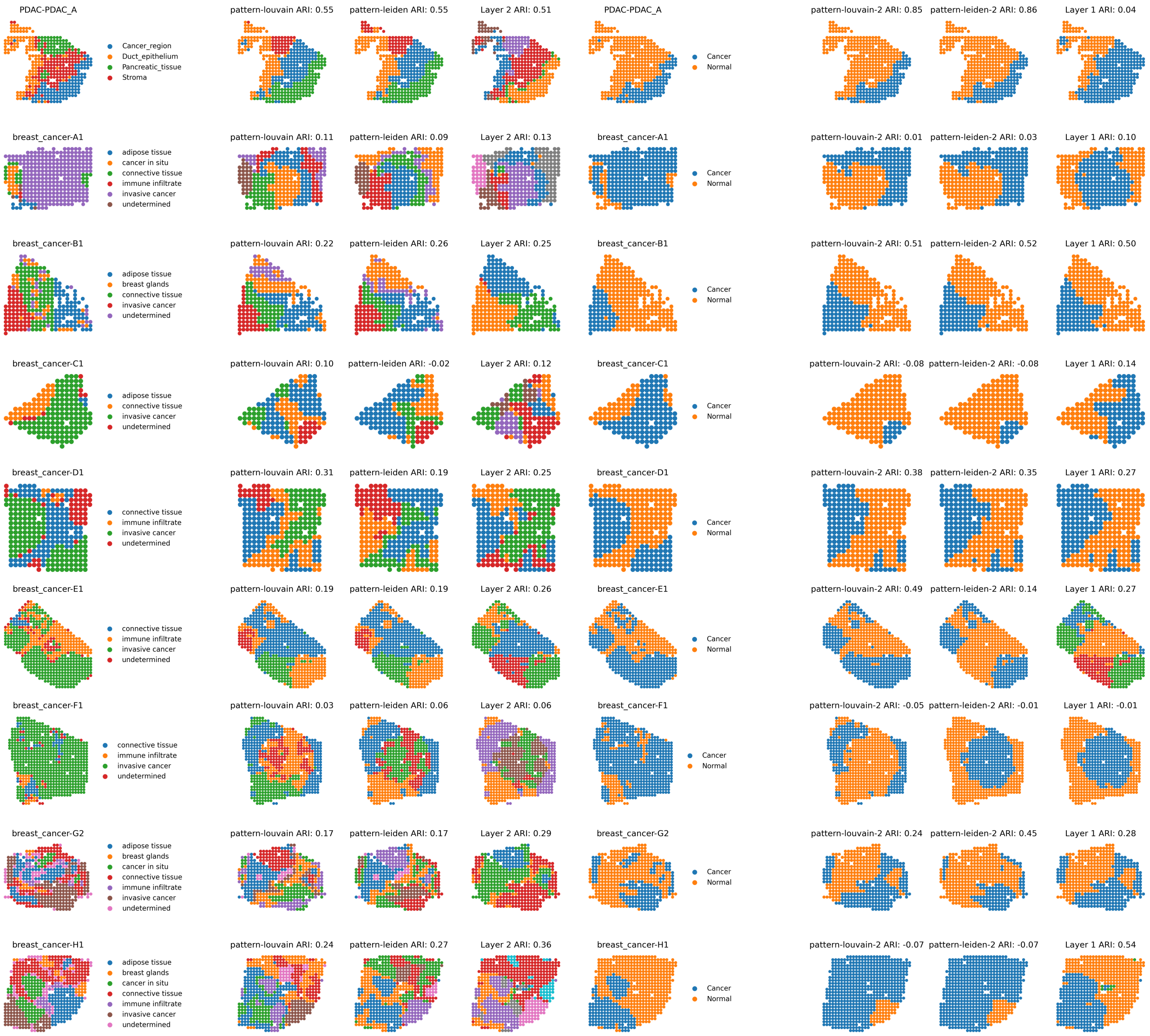

Supplementary Figure 14: Overview of clustering results on spatial transcriptome samples.
The first column shows the cell type annotations of each sample. The second and third columns show Louvain and Leiden clustering results using SVG patterns. The fourth column shows the results of layer 2 in CeiTEA. The fifth column shows the cancer-TME annotations of each sample. The sixth and seventh columns show Louvain and Leiden clustering results using SVG patterns. The eighth column shows the results of layer 1 in CeiTEA.

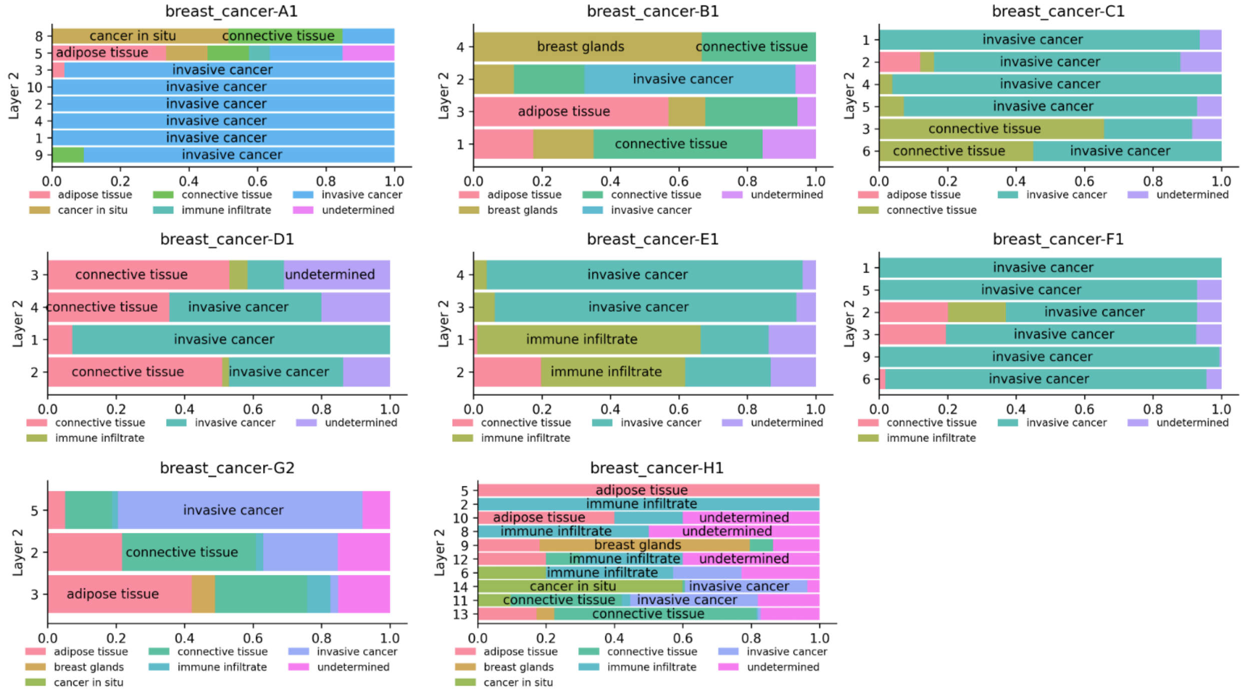

Supplementary Figure 15: Cell type compositions in Layer 2 clusters from the BC dataset.
Cell type compositions in Layer 2 clusters from each sample in the BC dataset. Clusters having a cell type with a majority of over 30% are displayed.

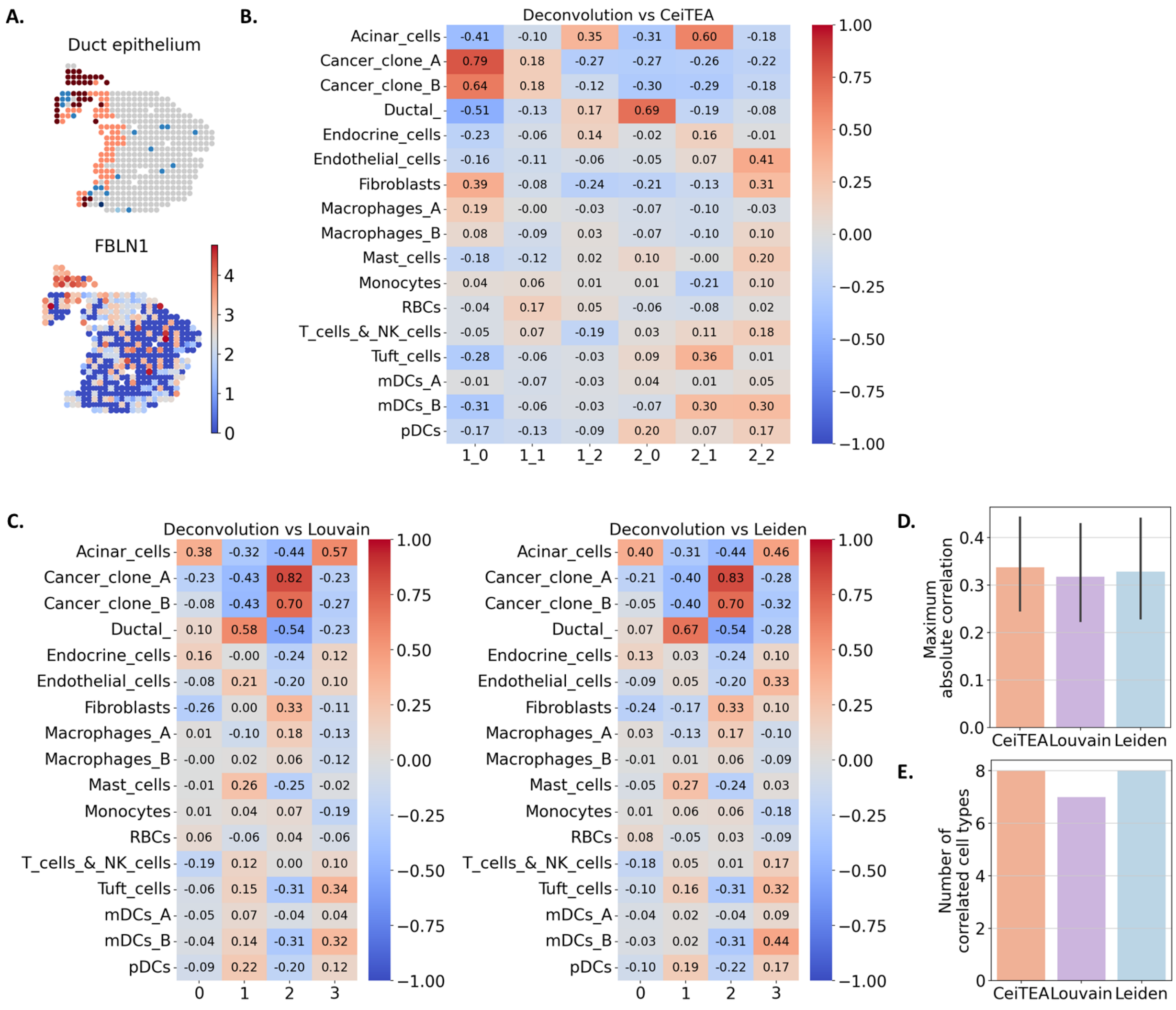

Supplementary Figure 16: Additional results on the PDAC dataset.
A. Top: Spatial distribution and contained Layer 2 clusters in the duct epithelium region; bottom: marker gene *KRT19* of cluster 2_4. **B.** Correlation heatmap between cell type deconvolution results and Layer 2 labels. **C.** Correlation heatmaps between cell type deconvolution results and Louvain (left) and Leiden (right) labels. **D.** Barplots of maximum absolute correlation per cell type across three methods, and number of correlated cell types (with Pearson $r \geq 0.3$) across three methods.

References

[1] Ulrike von Luxburg. A Tutorial on Spectral Clustering. 2007. doi: 10.48550/ARXIV.0711.0189.
